# Decrease of energy spilling in *Escherichia coli *continuous cultures with rising specific growth rate and carbon wasting

**DOI:** 10.1186/1752-0509-5-106

**Published:** 2011-07-05

**Authors:** Kaspar Valgepea, Kaarel Adamberg, Raivo Vilu

**Affiliations:** 1 Tallinn University of Technology, Department of Chemistry, Akadeemia tee 15, 12618 Tallinn, Estonia; 2 Competence Centre of Food and Fermentation Technologies, Akadeemia tee 15b, 12618 Tallinn, Estonia; 3 Tallinn University of Technology, Department of Food Processing, Ehitajate tee 5, 19086 Tallinn, Estonia

## Abstract

**Background:**

Growth substrates, aerobic/anaerobic conditions, specific growth rate (μ) etc. strongly influence *Escherichia coli *cell physiology in terms of cell size, biomass composition, gene and protein expression. To understand the regulation behind these different phenotype properties, it is useful to know carbon flux patterns in the metabolic network which are generally calculated by metabolic flux analysis (MFA). However, rarely is biomass composition determined and carbon balance carefully measured in the same experiments which could possibly lead to distorted MFA results and questionable conclusions. Therefore, we carried out both detailed carbon balance and biomass composition analysis in the same experiments for more accurate quantitative analysis of metabolism and MFA.

**Results:**

We applied advanced continuous cultivation methods (A-stat and D-stat) to continuously monitor *E. coli *K-12 MG1655 flux and energy metabolism dynamic responses to change of μ and glucose-acetate co-utilisation. Surprisingly, a 36% reduction of ATP spilling was detected with increasing μ and carbon wasting to non-CO_2 _by-products under constant biomass yield. The apparent discrepancy between constant biomass yield and decline of ATP spilling could be explained by the rise of carbon wasting from 3 to 11% in the carbon balance which was revealed by the discovered novel excretion profile of *E. coli *pyrimidine pathway intermediates carbamoyl-phosphate, dihydroorotate and orotate. We found that carbon wasting patterns are dependent not only on μ, but also on glucose-acetate co-utilisation capability. Accumulation of these compounds was coupled to the two-phase acetate accumulation profile. Acetate overflow was observed in parallel with the reduction of TCA cycle and glycolysis fluxes, and induction of pentose phosphate pathway.

**Conclusions:**

It can be concluded that acetate metabolism is one of the major regulating factors of central carbon metabolism. More importantly, our model calculations with actual biomass composition and detailed carbon balance analysis in steady state conditions with -omics data comparison demonstrate the importance of a comprehensive systems biology approach for more advanced understanding of metabolism and carbon re-routing mechanisms potentially leading to more successful metabolic engineering.

## Background

*Escherichia coli *exerts a very different gene and protein expression profile under different growth substrates [1], aerobic/anaerobic conditions [2] etc. Specific growth rate (μ) has been shown to be one of the most definite parameters influencing *E. coli *cell physiology as shown by studies of cell size [3,4], biomass composition [5-7], energy metabolism [5,8], transcriptome and proteome [9-11] etc..

To gain insights into the regulation and control mechanisms behind these different phenotype properties, it is useful to know carbon flow patterns in the metabolic network. A widely used tool to calculate quantitative flux values and thereby describe the carbon flow is metabolic flux analysis (MFA). Essentially, MFA calculations need a metabolic network with its stoichiometry, biomass amount and composition, measured steady state carbon influx and outflow-usually as CO_2 _and by-products. Flux distributions can also be calculated for batch cultures-however, the obtained values have to be considered with great care as the physiological state of cells is constantly changing during growth (*e.g*. μ, by-product production rates). Therefore, MFA is generally carried out with steady state input data from chemostat cultures which provide reproducible and strictly defined physiological state of cells [7,9,12-14].

*E. coli *mainly uses the consumed carbon for biomass formation and substantial amount of it goes to CO_2 _production. The flux (loss) of carbon to CO_2 _is closely associated with energy generation (spilling). Carbon usage for biomass synthesis and CO_2 _in the carbon balance can be directly measured *in situ *[7,13-15]. However, a notable amount of carbon is lost to many by-products excreted by the cells. The main by-product for most *E. coli *strains in aerobic cultivations is acetic acid [11,13,16]. In addition, accumulation of other compounds such as lactate, formate, pyruvate, ethanol etc. has been observed [7,13,17]. Although excretion of many other compounds besides 'well-known' ones *e.g*. pyrimidine pathway intermediates has been detected [9,18,19], no attention has been drawn on carefully measuring these carbon wasting substances in MFA studies, meaning also that the used metabolic network could be not completely accurate. This can result in a non-closed carbon balance subsequently leading to questionable conclusions. For instance, Taymaz-Nickerel *et al*. accounted a substantial amount of 'leftover carbon' in the carbon balance (7-13%) of *E. coli *continuous cultures to cells lysis which has not been observed before in the literature [7]. Comprehensive carbon balance analysis is, hence, essential for an accurate description of carbon flow and its regulation in the metabolic network under study.

Besides carbon inflow and outflow, biomass composition is another important input parameter in terms of MFA solutions also shown by sensitivity analysis [5]. However, rather than determining it in the same experiments, input values are usually taken from across the literature [7,9,12,15]. Since biomass composition varies under different growth conditions [5-7,15,20] obtaining its values from other studies, with various environmental conditions using various strains of *E. coli*, is another step in addition to a non-comprehensive carbon balance analysis that could possibly lead to distorted MFA results and drawing incorrect conclusions in terms of metabolic regulation. Therefore, we carried out both detailed carbon balance and biomass composition analysis in the same experiments to produce more accurate metabolic flux calculations. What is more, important metabolic switch points and regulation dynamics can be missed when using chemostat cultures. To continuously monitor the flux and energy metabolism dynamic responses to change in μ, we applied the accelerostat (A-stat) [4] continuous cultivation technique which has lately been demonstrated to produce a new regulation mechanism for overflow metabolism of acetate in *E. coli *[11] and an interesting growth efficiency strategy for *Lactococcus lactis *[21]. In addition to A-stat, dilution rate stat (D-stat) method [22] was used to study the effect of glucose-acetate co-utilisation capability on carbon wasting and metabolic flux patterns since this characteristic is proposed to be the key player in *E. coli *overflow metabolism [11].

In short, we detected a 36% reduction of ATP spilling in *E. coli *continuous cultures with increasing μ and carbon wasting under constant biomass yield (Y_XS_). We propose hypotheses about Y_XS _maintenance mechanisms and maximal growth limitations for *E. coli *K-12 MG1655. Furthermore, our study revealed novel carbon wasting profiles into pyrimidine pathway intermediates and acetate metabolism governed metabolic flux dynamics that are dependent on μ and glucose-acetate co-utilisation capability.

## Results

We carried out three replicate A-stat and four D-stat continuous cultivation experiments at various dilution rates with *E. coli *K-12 MG1655 which growth characteristics are described in detail in Valgepea *et al*. [11]. Carbon balance and biomass composition was carefully determined and the acquired data was used in MFA to obtain better understanding about carbon flow in the metabolic network.

### Metabolomic responses to rising μ in A-stat

**Figure 1:**
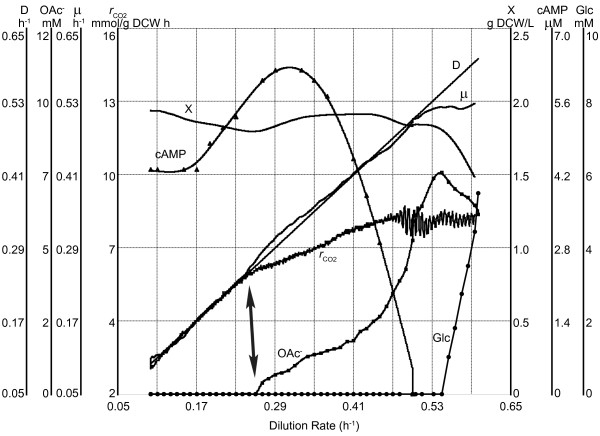
**Increasing dilution rate dependent *E. coli *K-12 MG1655 metabolism characterization in one A-stat cultivation**. D, dilution rate (h^-1^); X, biomass concentration (g dry cellular weight (g DCW)/L); μ, specific growth rate (h^-1^); *r*_CO2_, specific CO_2 _production rate (mmol/g DCW h); OAc^-^, acetate concentration (mM); Glc, glucose concentration (mM); cAMP, cyclic AMP concentration (μM). Arrow indicates the start of overflow metabolism. Start of vertical axes was chosen for better visualization. Reproduced by permission from Valgepea *et al*. [11].

**Figure 2:**
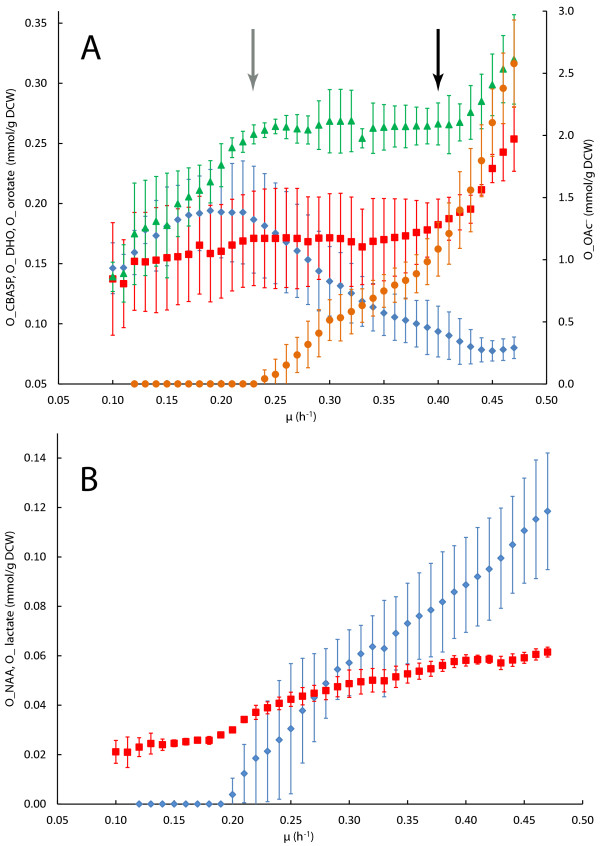
**Specific growth rate dependent dynamic carbon wasting profiles in three *E. coli *K-12 MG1655 A-stat cultivations**. μ, specific growth rate (h^-1^). Error bars represent standard deviation of triplicate A-stat experiments. A. Production of compounds per biomass (mmol/g DCW): CBASP, carbamoyl-aspartate (green triangle); DHO, dihydroorotate (blue diamond); orotate (red square); OAc^-^, acetate (orange circle). Grey arrow denotes acetate overflow switch with concomitant stop of DHO and CBASP increase whereas black arrow depicts faster acetate accumulation coupled induction of orotate and CBASP excretion. B. Production of compounds per biomass (mmol/g DCW): NAA, acetyl-aspartate (blue diamond); lactate (red square).

We detected a two-phase acetate accumulation profile in A-stat which started at μ = 0.27 ± 0.02 h^-1 ^(average ± standard deviation) (Figure 1 &2A). Faster accumulation of acetate that took place simultaneously with the abrupt decline in cAMP was witnessed after *E. coli *had reached maximum CO_2 _production at μ = 0.46 ± 0.02 h^-1 ^(Figure 1). Additionally, we observed considerable excretion of pyrimidine pathway intermediates during increase of μ in three phases (Figure 2A). Dihydroorotate (DHO) and carbamoyl-aspartate (CBASP) accumulated increasingly up to the start of acetate overflow. After overflow switch, DHO started to decline whereas orotate and CBASP levelled off until their levels started to rise again simultaneously (Figure 2A) with the sharp decrease of cAMP and faster accumulation of acetate (Figure 1). Interestingly, in addition to lactate excretion, we observed increased accumulation of a compound once noted in the literature-acetyl-aspartate (NAA)-with rising μ (Figure 2B).

### Metabolic flux dynamics with increasing μ

**Figure 3:**
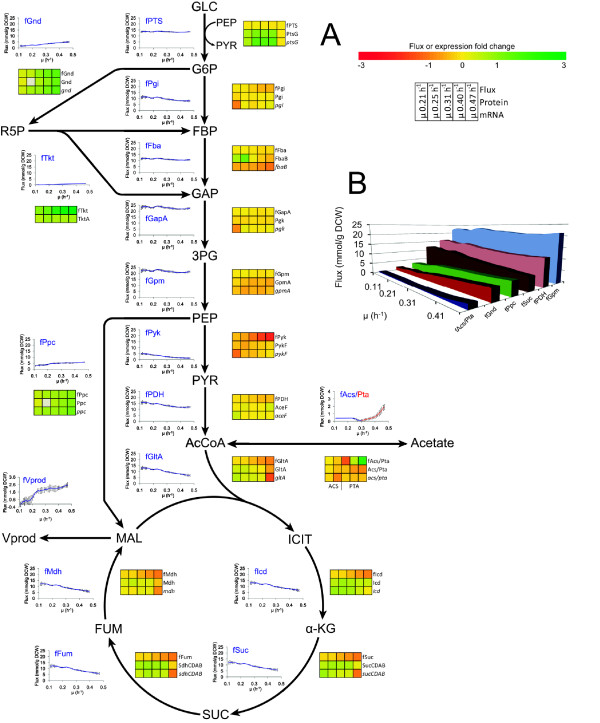
***E. coli *K-12 MG1655 central carbon metabolism flux, protein and mRNA dynamics with rising specific growth rate in three A-stat cultivations**. μ, specific growth rate (h^-1^). A. Flux or expression fold change (log_2_) is calculated for each μ compared to μ = 0.10 h^-1^; grey box depicts missing value; Acs flux was switched to Pta after μ = 0.31 h^-1 ^in MFA since acetate excretion exceeds its production accompanying biosynthesis. Protein and mRNA data taken from Valgepea *et al*. [11]. Error bars represent standard deviation of triplicate A-stat experiments. B. Selected glycolysis, TCA cycle, PPP, gluconeogenesis and acetate related fluxes.

**Figure 4:**
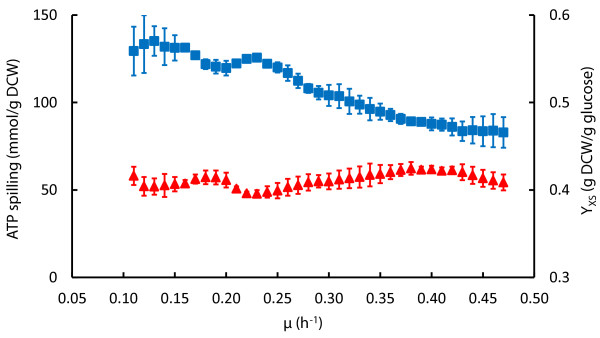
**Specific growth rate dependent ATP spilling and biomass yield in three *E. coli *K-12 MG1655 A-stat cultivations**. μ, specific growth rate (h^-1^); ATP spilling, non-growth associated ATP production (blue square); Y_XS_, biomass yield as gram dry cellular weight per gram consumed glucose (red triangle). Error bars represent standard deviation of triplicate A-stat experiments.

We used data from μ dependent detailed carbon balance and biomass composition analysis to carry out MFA for describing the carbon flow in our metabolic network. Biomass composition was dependent on μ (Table S1 in Additional file 1) and its incorporation into MFA calculations was important as shown by up-to 15% difference in flux values compared to using constant biomass composition at different μ (Table S2 in Additional file 1). Our simplified metabolic network (Figure S1 in Additional file 2) consisted of three main pathways-glycolysis, pentose phosphate pathway (PPP), tricarboxylic acid (TCA) cycle-, a part of pyrimidine pathway (to include CBASP, DHO, orotate) and NAA synthesis reaction with 50 fluxes, 22 metabolites taking into account ATP, NADH and NADPH stoichiometry (see Methods and Additional file 2 for details). MFA results for both A-stat and D-stat experiments are given in Additional file 1 (Tables S3-5). Start of acetate overflow triggered reduction of TCA cycle fluxes (Figure 3) also seen by decline of the proportion of CO_2 _and NADH produced by TCA cycle (Additional file 3). This subsequently led to induction of PPP fluxes, reduction of glycolysis (Figure 3) and ATP produced from it (Additional file 4). An important central carbon metabolism branch point flux, pyruvate dehydrogenase reaction, reached its maximum throughput at μ = 0.42 h^-1 ^with concomitant slight increase in glycolysis fluxes resulting in accelerated carbon wasting into by-products (Figure 2 &3). Decrease of Pyk and increase of Ppc and Vprod (excess carbon outflow flux from oxaloacetate in our model) fluxes until acetate accumulation shows that some up-taken carbon was still in excess and excreted as Vprod through Ppc flux (Figure 3). mRNA and protein μ dependent expression dynamics are illustrated as heat maps for all the central carbon metabolism fluxes, see Figure 3A. Correlations between mRNA, protein and flux fold changes and possible metabolic regulation mechanisms will be discussed in a subsequent article. Surprisingly, a strong 32% reduction of ATP spilling (non-growth associated ATP production) was observed after disruption of the PTA-ACS cycle [11] resulting in acetate overflow switch (Figure 4). Reduction of ATP spilling was witnessed since the futile PTA-ACS cycle was included into the model network, see Discussion. Overall ATP production rate increased with μ rise but changed its slope after overflow switch (Additional file 4).

### Carbon balance analysis in A-stat

**Figure 5:**
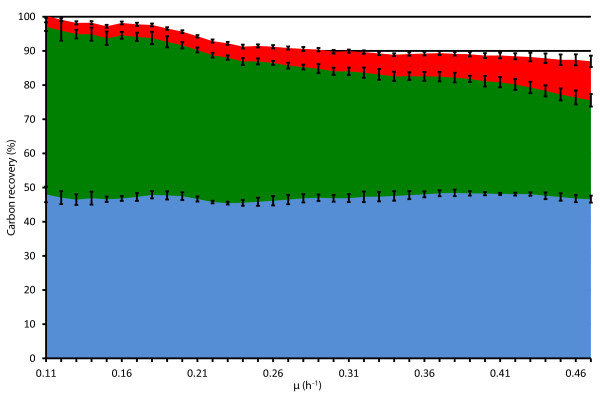
**Specific growth rate dependent carbon balance in three *E. coli *K-12 MG1655 A-stat cultivations**. μ, specific growth rate (h^-1^). Carbon recovery % in carbon balance into: biomass (blue); CO_2 _(green); sum of carbon wasting into acetate, CBASP, DHO, lactate, NAA, orotate (red). Error bars represent standard deviation of triplicate A-stat experiments.

**Figure 6:**
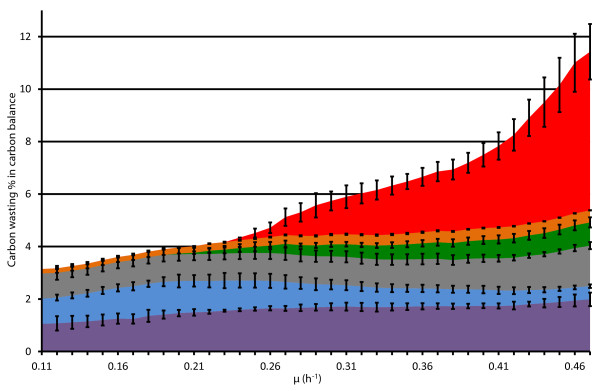
**Specific growth rate dependent dynamic carbon wasting profiles in three *E. coli *K-12 MG1655 A-stat cultivations**. μ, specific growth rate (h^-1^). Carbon wasting % in carbon balance into: acetate (red); lactate (orange); NAA, acetyl-aspartate (green); orotate (grey); DHO, dihydroorotate (blue); CBASP, carbamoyl-aspartate (violet). Error bars represent standard deviation of triplicate A-stat experiments.

Detailed carbon balance analysis in A-stat showed that most of carbon was used for biomass generation and its proportion relative to CO_2 _production increased with rising μ (Figure 5). It can be seen that the carbon balance was not fully closed especially at higher μ which points to loss of carbon into some other not detected compounds. Carbon wasting into by-products increased from 3 to 11% in the carbon balance within the studied μ range (Figure 5 &6). Acetate quickly became the main excreted compound by amount after overflow switch.

### Carbon wasting and metabolic flux dynamics in D-stat

**Figure 7:**
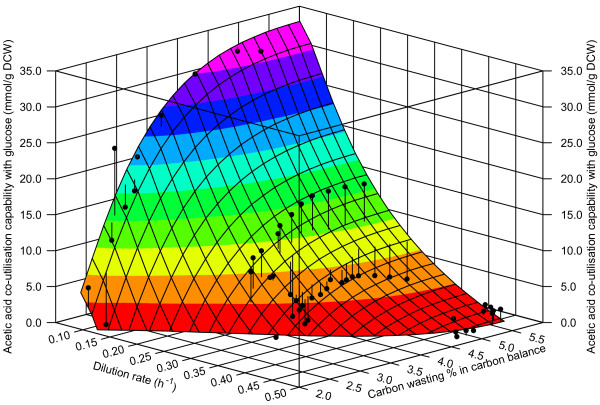
**3D surface describing the relationship between dilution rate, glucose-acetate co-utilisation capability and carbon wasting % in carbon balance in four *E. coli *K-12 MG1655 D-stat cultivations**. Black dots represent real data points of D-stat experiments at D = 0.1; 0.24; 0.3; 0.45 h^-1^. The surface was drawn based on good regression and illustrative characteristics.

In addition to A-stat, we carried out four D-stat experiments at various dilution rates to study the effect of glucose-acetate co-utilisation capability on carbon wasting and metabolic flux patterns. Capability of *E. coli *to co-utilise acetate simultaneously with glucose was strongly repressed with increasing dilution rates (Figure 7). We observed that carbon wasting patterns into orotate, DHO, CBASP, NAA and lactate changed under different co-utilisation properties (Table S5 in Additional file 1). It is interesting to note that the percentage of overall carbon wasting (sum of orotate, DHO, CBASP, NAA and lactate) in the carbon balance was similar (*ca *5.5%) at the studied dilution rates under very different maximal glucose-acetate co-utilisation capability values (Figure 7). As expected, MFA calculations with D-stat data revealed that acetate consumption fluxes together with glyoxylate shunt and gluconeogenesis were higher under higher glucose-acetate co-utilisation values (Table S5 in Additional file 1). Concomitantly, glycolysis fluxes were down-regulated at lower co-utilisation conditions similarly with pyruvate dehydrogenase.

### A-stat comparison with chemostat

A-stats have been shown to produce quantitatively comparable results with chemostats at the level of *E. coli *and *Lactococcus lactis *major growth characteristics (*e.g*. Y_XS_, by-product production per biomass) [11,21] and *E. coli *transcriptome [11]. Our detailed carbon balance analysis further confirmed that A-stat and chemostat data are quantitatively comparable (Table 1) which enables to use quasi steady state data from A-stat for steady state modeling calculations.

**Table 1:** A-stat and chemostat non-CO_2 _by-product production comparison

	μ = 0.10 h^-1^		μ = 0.24 h^-1^		μ = 0.30 h^-1^		μ = 0.45 h^-1^	
	**Chemostat**	**A-stat**	**Chemostat**	**A-stat**	**Chemostat**	**A-stat**	**Chemostat**	**A-stat**
	
O_CBASP_	0.188	0.139 ± 0.047	0.089	0.218 ± 0.018	0.195	0.268 ± 0.026	0.188	0.299 ± 0.026
O_DHO_	0.131	0.146 ± 0.021	0.117	0.181 ± 0.043	0.091	0.135 ± 0.029	0.065	0.078 ± 0.008
O_lactate_	ND	0.021 ± 0.005	0.034	0.041 ± 0.003	0.034	0.049 ± 0.006	0.046	0.059 ± 0.002
O_NAA_	ND	ND	0.006	ND	0.034	0.057 ± 0.013	0.069	0.111 ± 0.021
O_orotate_	0.091	0.137 ± 0.047	0.193	0.171 ± 0.041	0.115	0.171 ± 0.037	0.344	0.229 ± 0.012

Unit is mmol/g DCW. A-stat values represent the average from three independent experiments and standard deviation follows the ± sign. Chemostat values from one experiment. Refer to text for abbreviations. ND, not detected.

## Discussion

We applied a systems biology approach to study *E. coli *metabolic flux dynamics and possible growth limiting factors. Detailed carbon balance and biomass composition analysis were carried out in A-stat and D-stat cultures to examine the dynamic responses of metabolic fluxes and energy metabolism to change of μ and glucose-acetate co-utilisation capability. A simplified MFA was conducted to map the carbon flow through central carbon metabolism.

We detected a two-phase acetate accumulation profile in A-stat which started at μ = 0.27 ± 0.02 h^-1 ^(Figure 1). After linear increase, acetate probably starts to accumulate exponentially because of total repression of acetate consuming enzyme, acetyl-CoA synthetase, by carbon catabolite repression [11]. It became clear from MFA calculations that acetate excretion plays an important role in overall flux patterns and ATP metabolism. Firstly, start of acetate excretion reduces carbon flow from the PTA-ACS cycle to acetyl-CoA and central metabolism triggering reduction of TCA cycle fluxes (Figure 3) that can be also seen by decline of the proportion of CO_2 _and NADH produced by the TCA cycle (Additional file 3). This was paralleled with induction of PPP, possibly for NADPH regeneration, and reduction of glycolysis (Figure 3). These shifts have been observed in chemostat cultures as well [9,12-14,23]. Secondly, disruption of the PTA-ACS node resulting in acetate overflow strongly reduced ATP spilling (non-growth associated ATP production) which declined 32% with increasing of μ (Figure 4). What is more, slope of the overall ATP production rate changed right after overflow switch (Additional file 4). It can be concluded from the latter that acetate metabolism is one of the major regulating factors of central carbon metabolism which is in good agreement with abundance of literature data.

Decrease in ATP spilling (32%) after overflow switch in A-stat was shown by MFA calculations (Figure 4) and decline of the ATP-spending PTA-ACS node throughput by acetate excretion (Figure 2A). This response in energy metabolism was detected in this study since the futile PTA-ACS cycle was included into the model network. Additionally, change in ATP production rate was also seen with increasing μ (Additional file 4). We have to note that the possibility of our ATP calculations being distorted due to carbon imbalance at higher specific growth rates (Figure 5) cannot be excluded which could lead to underestimation of ATP production. However, this seems rather unlikely since CO_2 _measurement precision is constant for all μ. Furthermore, theoretically no other pathway besides the TCA cycle cannot by far produce enough energy under these carbon imbalance conditions so that decrease of ATP spilling would not be observed. We additionally have to point out that our calculations could also overestimate ATP production since a theoretical ratio for ATP generation efficiency in oxidative phosphorylation (P/O = 2) was chosen which can be higher than the *in vivo *value. In any case, the P/O ratio *per se *does not affect the main conclusions of the manuscript since the ratio between ATP spilling and its overall production is independent from the P/O ratio value.

Decrease in ATP spilling (40 mmol/g dry cellular weight (DCW)) might indicate increase of Y_XS_, however, it remained constant in our experiments (Figure 4). This apparent discrepancy between the decrease in ATP spilling and constant Y_XS _(Figure 4) could be explained by the fact that carbon wasting increases from 3 to 11% with rising μ (Figure 6) as follows. As the acetate synthesis/assimilation PTA-ACS is a futile cycle, an equivalent amount of ATP to acetate is concomitantly wasted with production and re-consumption of acetate. Therefore, accumulation of acetate likely triggers a 32% decline of ATP spilling (Figure 4) since re-consumption of acetate (wasting 1 molecule of ATP) decreases with rising μ after overflow switch. This energy save is, however, counteracted by the increase of carbon wasting in the carbon balance from 3 to 11% which results in a constant Y_XS_. However, *E. coli *might possess additional mechanisms to maintain a constant Y_XS _under increasing carbon wasting conditions during μ increase.

In addition to metabolic flux dynamics, we described novel carbon wasting profiles in *E. coli *K-12 MG1655 into pyrimidine pathway intermediates orotate, DHO, CBASP, and NAA with rising μ (Figure 2) and under various glucose-acetate co-utilisation capabilities (Table S5 in Additional file 1). Excretion of orotate [18,19], DHO [19], CBASP [19] and NAA [9] by *E. coli *has been noted before. Accumulation of the pyrimidine pathway compounds-orotate, DHO and CBASP-can be explained by the *E. coli *K-12 MG1655 genotype. This specific strain is prone to pyrimidine starvation due to a *rph *frameshift mutation leading to low *pyrE *(encodes PyrE protein which catalyses orotate conversion into orotidine-5-phosphate) expression [24] which could possibly lead to accumulation of precursor molecules which all the latterly mentioned compounds are (Additional file 5). Excretion of a considerable amount of CBASP, DHO, orotate and NAA besides acetate shows that overflow metabolism actually consists of more products than acetate, as generally believed. Detailed by-product measurements enabled us to precisely detect carbon outflow routes for MFA calculations which usually are taken into account predictively either from pyruvate, oxaloacetate, α-ketoglutarate or other potential precursors. For instance, if these product outflows will be excluded from MFA and carbon wasting predicted as pyruvate outflow, pyruvate dehydrogenase, TCA cycle and PEP carboxylase fluxes would deviate by 11, 24 and 60%, respectively, at μ = 0.47 h^-1 ^from the values calculated by our model (Table S6 in Additional file 1). Although the absolute amount of these excreted substances in the carbon balance is not substantial (less than 5%), linking their accumulation dynamics to μ (or metabolic routes) is relevant for acknowledging the potential imbalance between pyrimidine metabolism, carbon re-consumption and ATP spilling.

DHO and CBASP accumulated increasingly up to the start of acetate overflow (Figure 2A). After overflow switch, DHO started to decline whereas orotate and CBASP levelled off suggesting that carbon flow from the PTA-ACS cycle to acetyl-CoA and central metabolism declines indicated by carbon loss to excreted acetate and decreasing TCA cycle flux patterns (Figure 2A &3). Finally, orotate and CBASP levels started to increase again simultaneously (Figure 2A) with the sharp decrease of cAMP and faster accumulation of acetate (Figure 1). Firstly, this rise could be explained by the high demand for RNA synthesis at higher μ which leads to precursor molecule accumulation because of the low *pyrE *expression. On the other hand, pyruvate dehydrogenase flux reached its maximum at μ = 0.42 h^-1 ^with concomitant slight increase in glycolysis fluxes that subsequently resulted in accelerated carbon wasting (Figure 3). These observations demonstrate a strong link between overflow metabolism of acetate and carbon wasting into other products.

We validated and quantified, for the first time to our knowledge, excretion of NAA which levels constantly increased with μ in A-stat experiments (Figure 2B). Neither has NAA yet been registered in EcoCyc Ver 15.0 [25] nor there exists a protein catalysing its synthesis for *E. coli *K-12 MG1655 in KEGG Release 58.0 [26]. Based on homology analysis with the available aspartate N-acetyltransferase protein sequences (human and mouse), we propose that a predicted acetyltransferase YjgM could catalyse the formation of NAA in *E. coli *(Additional file 6). This is supported by the fact that YjgM is expressed within the studied μ range [11]. We hypothesise that since oxaloacetate is over-produced in the TCA cycle and this excess carbon cannot be shunted towards PPP because of the discovered 'CBASP-DHO-orotate' bottleneck, the excess carbon is excreted as NAA.

To our knowledge, dilution rate dependent carbon wasting profiles in terms of two-substrate (glucose and acetic acid) co-utilisation has not been studied before. We found that carbon wasting patterns are dependent not only on μ as shown by A-stat, but also on glucose-acetate co-utilisation capability (Figure 7). This finding could be useful for bioprocess development since mixed-substrate growth is commonly used there and loss of carbon is unwanted. What is more, overall carbon wasting into the carbon wasting products in the carbon balance was similar (*ca *5.5%) under very different maximal glucose-acetate co-utilisation capability values (Figure 7). The latter implies that the quantitative carbon flow through the carbon wasting pathways does not depend on the consumption of additional (to glucose) carbon sources, such as acetate in our case.

Overall carbon wasting in the carbon balance increased with rising μ (Figure 5 &6). Carbon wasting to other substances than acetate *e.g*. orotate, DHO, CBASP, NAA may be caused by an imbalance between the supply of precursors, intermediates of central metabolic network and insufficient use of them for the synthesis of biomass monomers and macromolecules which could be the result of energy limitations. Levels of metabolites from the upper part of energy generating glycolysis, fructose-1,6-bisphosphate and glyceraldehyde-3-phosphate, and TCA cycle components increased with the rise in μ [11], indicating no limits in energy supply at these steps. However, there might be competing futile cycles wasting ATP, and leading to energy limitations, for instance in glycolysis [27] and the PTA-ACS cycle proposed by us [11]. In addition to the pyrimidine synthesis pathway compounds-orotate, DHO and CBASP-, we observed TCA cycle intermediates α-ketoglutarate and isocitrate accumulation, and decline in intracellular ribose-5-phosphate and erythrose-4-phosphate metabolite levels, which are also intermediates of pyrimidine synthesis [11]. This might result in growth limitation by RNA synthesis and ribosome assembly, especially at higher μ. The latter is supported by the fact about the genotype of *E. coli *K-12 MG1655 (*rph *frameshift mutation leading to low *pyrE *expression) which leads to pyrimidine starvation as described above. The latter proposal is in agreement with RNA concentration measurements which showed that RNA amount increased together with μ until 0.40 h^-1 ^after which it levelled off (Table S1 in Additional file 1). The possible limitation of maximal μ by RNA synthesis and carbon wasting due to the *E. coli *K-12 MG1655 genotype revealed by this study proposes a way how to increase maximal μ and Y_XS _which is relevant for the biotechnology industry. Furthermore, all the data referred above show that the details of the regulatory mechanisms of cellular growth need further studying using comprehensive systems biology approaches.

## Conclusions

In this work, we described a novel carbon wasting strategy to pyrimidine pathway intermediates and acetate metabolism governed metabolic flux dynamics in *E. coli *continuous cultures dependent on μ and glucose-acetate consumption capability. More importantly, MFA calculations with actual biomass composition revealed a 36% reduction of ATP spilling with increasing μ and carbon wasting to non-CO_2 _by-products under constant Y_XS_. Our model calculations with actual biomass composition and detailed carbon balance analysis in steady state conditions with -omics data comparison demonstrate the importance of a comprehensive systems biology approach for more advanced understanding of carbon re-routing mechanisms.

## Methods

### Bacterial strain, medium and continuous cultivation conditions

*E. coli *K-12 MG1655 (λ-F-*rph-1Fnr+*; Deutsche Sammlung von Mikroorganismen und Zellkulturen (DSMZ), DSM No.18039) was used in A-stat and D-stat experiments under the following conditions: temperature 37°C, pH 7, agitation speed of 800 rpm, and aerobic conditions (air flow rate 150 ml/min). Three A-stat cultivations were performed with acceleration rate 0.01 h^-2 ^and four D-stat experiments at dilution rates 0.10; 0.24; 0.30; 0.45 h^-1^. A detailed description of medium and cultivation conditions in these experiments has been reported previously [11]. In short, defined minimal medium with 4.5 g/L α-(D)-glucose was used in A-stat experiments. The latter was also used in D-stat experiments as the main cultivation medium, additionally the main medium was supplemented with acetic acid and prepared as follows: 300 ml medium was withdrawn from the main cultivation medium and supplemented with 3 ml of glacial acetic acid (99.9%).

### Analytical methods

Biomass concentration was determined gravimetrically as dry cellular weight (expressed as DCW) described by Nahku *et al*. [10].

Samples of culture medium for extracellular metabolome analysis were centrifuged at 14,000 × *g *for 5 min, supernatant were collected and analysed immediately or stored at -20°C until analysis. Glucose and organic acids were analysed by HPLC (Alliance2795 system, Waters, Milford, MA) using a BioRad HPX-87H Aminex ion-exclusion column connected to RI and UV detectors (35°C, flow rate 0.6 ml/min). The column was eluted with 4.1 mM sulphuric acid for glucose, CBASP, lactate, orotate and with 26.5 mM formic acid for acetate, DHO and NAA analysis.

### Metabolic flux analysis (MFA)

Simplified metabolic network of *E. coli *K-12 MG1655 for MFA was reconstructed taking into account main metabolic pathways-glycolysis, PPP, TCA cycle-, one pathway from pyrimidine metabolism (to include CBASP, DHO, orotate) and NAA synthesis reaction (Figure S1 in Additional file 2). Our network involved only fluxes (reactions) between branching points (metabolites) whereas linear pathway chains were lumped together. Fully determined and calculable stoichiometric matrix consisted of 22 metabolites and 50 fluxes (24 dependent fluxes, one measured inflow, seven outflow fluxes and 18 calculated fluxes based on biomass composition and stoichiometries of biosynthetic pathways). Biomass composition was shown to be dependent on μ (Table S1 in Additional file 1). Cofactors ATP, NADPH and NADH were considered in calculations. Refer to Additional file 2 for detailed description for reaction stoichiometries and calculation of biomass composition dependent fluxes. MFA results for both A-stat and D-stat experiments are given in Additional file 1 (Table S3-5).

## Competing interests

The authors declare that they have no competing interests.

## Authors' contributions

KV, KA, and RV designed, guided and coordinated the project. KV performed the experiments, carried out analytics and data analysis. KV and KA were responsible for model calculations. KV drafted the manuscript. KA and RV helped in drafting the manuscript. All authors read and approved the manuscript.

## Supplementary Material

Additional file 1 **Specific growth rate dependent biomass composition and metabolic flux analysis results of triplicate *E. coli *K-12 MG1655 A-stat experiments**. Specific growth rate dependent *E. coli *K-12 MG1655 biomass monomer composition (Table S1); Effect of specific growth rate dependent biomass composition on MFA results (Table S2); MFA results for triplicate *E. coli *K-12 MG1655 A-stat experiments (Table S3); MFA average results and standard deviations of triplicate *E. coli *K-12 MG1655 A-stat experiments (Table S4); MFA results of four *E. coli *K-12 MG1655 D-stat experiments (Table S5); Effect of taking novel carbon wasting routes into account in *E. coli *K-12 MG1655 A-stat experiments' MFA (Table S6). Click here for file

Additional file 2 **Metabolic flux analysis**. Detailed description of model calculations with simplified metabolic flux analysis; Simplified metabolic network scheme of *E. coli *K-12 MG1655 (Figure S1). Click here for file

Additional file 3 ***E. coli *K-12 MG1655 proportion of CO_2 _and NADH production by TCA cycle with rising specific growth rate in three A-stat cultivations**. μ, specific growth rate (h^-1^). CO_2 _production (blue square); NADH production (red triangle). Error bars represent standard deviation of triplicate A-stat experiments. Click here for file

Additional file 4 **Specific growth rate dependent overall ATP production rate and proportion of ATP production by glycolysis in three *E. coli *K-12 MG1655 A-stat cultivations**. μ, specific growth rate (h^-1^). Overall ATP production (blue squares); ATP production by glycolysis (red triangle). Error bars represent standard deviation of triplicate A-stat experiments. Click here for file

Additional file 5 ***E. coli *K-12 MG1655 pyrimidine pathway *rph *frameshift mutation triggered accumulating precursor compounds**. Carbamoyl-P, carbamoyl-phosphate; CBASP, carbamoyl-aspartate; DHO, dihydroorotate; Oro-5P, orotidine-5-phosphate; TCA cycle, tricarboxylic acid cycle; PPP, pentose phosphate pathway; *pyrB*, aspartate carbamoyltransferase; *pyrC*, dihydro-orotase; *pyrD*, dihydro-orotate oxidase; *pyrE*, orotate phosphoribosyltransferase. Gene names are italicised. Click here for file

Additional file 6 **Homology analysis for *E. coli *acetyltransferase prediction**. BLAST results for homology analysis with *Homo sapiens *and *Mus musculus *aspartate N-acetyltransferases. Click here for file
