# Supplementary material for: Decrease of energy spilling in Escherichia coli continuous cultures with rising specific growth rate and carbon wasting
Source: BMC Syst Biol. 2011 Jul 5;5:106. doi: 10.1186/1752-0509-5-106 (PMC3149000; doi:10.1186/1752-0509-5-106)
Supplement: Additional file 2 — Metabolic flux analysis. Detailed description of model calculations with simplified metabolic flux analysis; Simplified metabolic network scheme of E. coli K-12 MG1655 (Figure S1). [file 1752-0509-5-106-S2.PDF]

Additional file 2. Metabolic flux analysis (MFA)

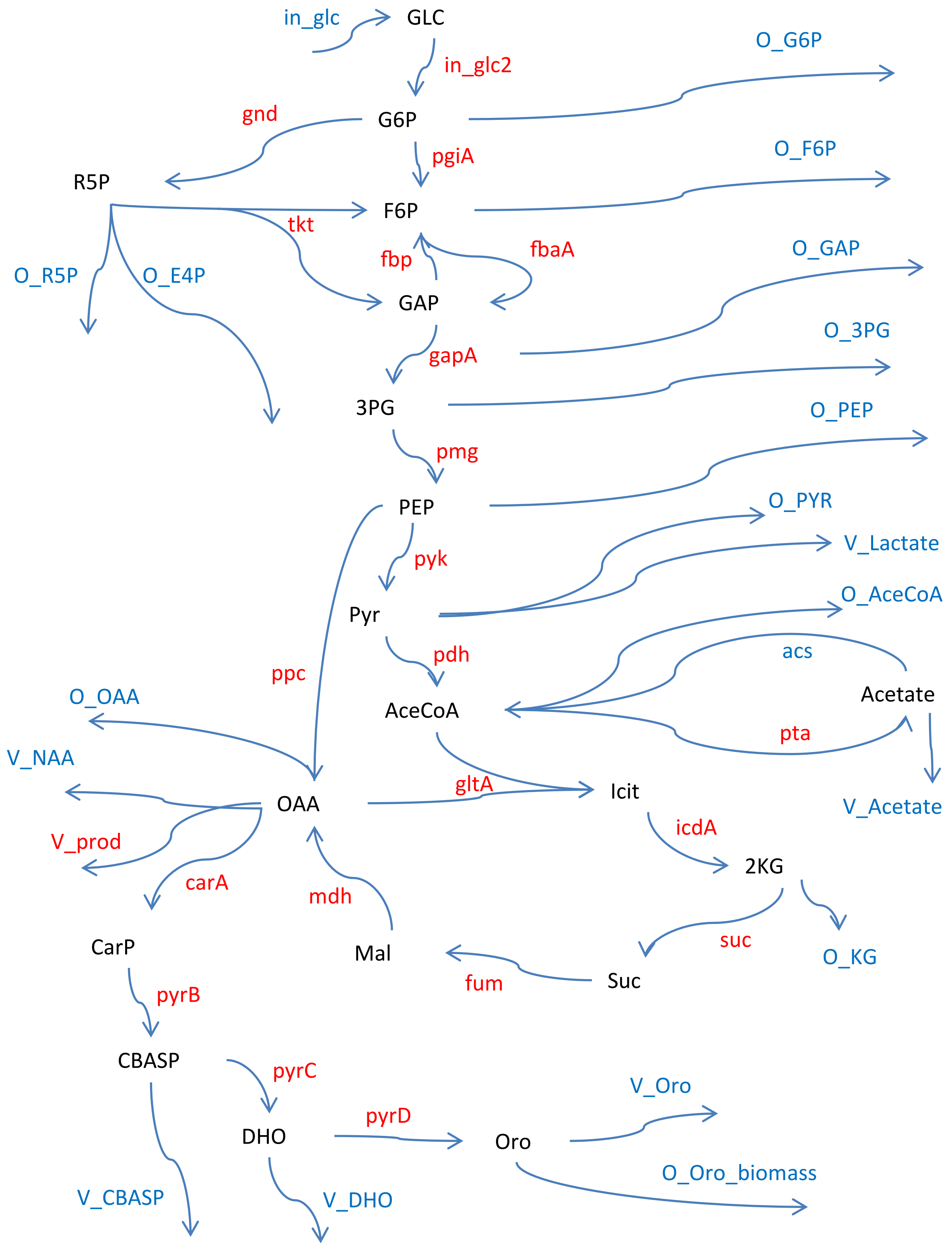

Figure S1
Simplified metabolic network of *E. coli* K-12 MG1655. Metabolites are indicated in black, calculated fluxes in red and measured fluxes in blue. G6P, glucose-6-phosphate; F6P, fructose-6-phosphate; R5P, ribose-5-phosphate; GAP, glyceraldehydephosphate; 3PG, 3-phosphoglycerate; PEP, phosphoenolpyruvate; Pyr, pyruvate; AceCoA, acetyl-CoA; Icit, isocitrate; 2KG, 2-oxoglutarate; Suc, succinate; Mal, malate; OAA, oxaloacetate; CarP, carbamoyl-phosphate; CBASP, carbamoyl-aspartate; DHO, dihydroorotate; Oro, orotate; Oro_biomass, orotate to biomass (pyrimidine synthesis); NAA, acetyl-aspartate.

24 dependent fluxes were:

1. In_glc2: Glc_ext + ATP = G6P
2. gnd: G6P = 2 NADPH + R5P + CO_2
3. tkt: 3 R5P = 2 F6P + GAP
4. pgiA: G6P = F6P
5. fbaA: F6P + ATP = 2 GAP
6. gapA: GAP = NADH + ATP + 3PG
7. pmg: 3 PG = PEP
8. pyk: PEP = ATP + Pyr
9. pdh: Pyr = NADH + Ace-CoA + CO_2
10. pta: AceCoA = ATP + Acetate or acs: Acetate + 2 ATP = AceCoA
11. gltA: AceCoA + OAA = Icit
12. icdA: Icit = NADPH + 2 KG + CO_2
13. suc: 2 KG = ATP + NADH + Suc + CO_2
14. fum: Suc = Mal + quinol
15. mdh: Mal = NADH + OAA
16. ppc: PEP + CO_2 = OAA
17. carA: 3 ATP + CO_2 = CarP
18. pyrB: CarP + OXA + NADPH = CBASP
19. pyrC: CBASP = DHO
20. pyrD: DHO = Oro + quinol
21. vNADH: NADH = 2 ATP
22. Vquin: quinol = ATP
23. ngATP: ATP =
24. V_prod : OAA =

Note: acs flux was switched to pta after $\mu = 0.31\ h^{-1}$ in MFA since acetate excretion exceeds its production accompanying biosynthesis. vNADH characterizes ATP production in respiratory chain if P/O = 2. ngATP characterizes non-growth associated ATP spilling if biomass components are synthesized once. V_prod characterizes carbon outflow that was not identified experimentally.

In case of D-stat experiments where *E. coli* co-utilised acetic acid with glucose the following two reactions were substituted (since these are active under growth on acetic acid): suc to aceAB (aceAB: AceCoA + Icit = Suc + Mal); ppc to pck (pck: OAA + ATP = PEP + CO_2).

8 measured fluxes were:

1. in_GLC: = GLC_ext
2. V_Lactate: Pyr + NADH =
3. V_Acetate: Acetate =
4. V_NAA: OAA + AceCoA + NADPH =
5. V_CBASP: CBASP =
6. V_DHO: DHO =
7. V_Oro: Oro =
8. V_CO_2: CO_2 =

18 calculated fluxes were:

1. O1: G6P =
2. O2: F6P =
3. O3: R5P =
4. O4: 2R5P = F6P

5. O5: GAP =
6. O6: 3PG =
7. O7: PEP =
8. O8: PYR =
9. O9: Ace-CoA =
10. O10: OAA =
11. O11: 2KG =
12. O12: O_Oro_biomass =
13. Onadh: = NADH
14. Onadph: NADPH =
15. Osynth: ATP =
16. Opolym: ATP =
17. mATP: ATP =
18. acetrans: 2ATP = AceCoA

Note: acetrans characterizes conversion of acetate (produced during arginine, cysteine and methionine synthesis) to AceCoA by ACS.

O-fluxes (biomass fluxes) were calculated based on biomass monomer composition and reaction stoichiometries from central metabolites to monomers (20 amino acids, 8 nucleotides, 6 fatty acids and 3 mono-saccharides, see Table S1 in Additional file 1). Amino acid (using AccQTaq Ultra pre-column derivatization kit and UPLC (Waters) according to the manufacturer's instructions from hydrolyzed culture samples), fatty acids (using saponification by KOH and fatty acid quantification by UPLC as in Špitsmeister *et al.*[1]) and total RNA (using Qiagen RNA quantification kit) were experimentally measured from biomass. DNA and ash content in biomass was taken from Neidhardt *et al.*, (1987) [2] and residual water in dry biomass was estimated as 8 %. Polysaccharide content was calculated as residual of above mentioned components. MFA results for both A-stat and D-stat experiments are given in Additional file 1 (Table S3–5).

Supplementary References

1. Špitsmeister M, Adamberg K, Vilu R: **UPLC/MS based method for quantitative determination of fatty acid composition in Gram-negative and Gram-positive bacteria.** *J Microbiol Methods* 2010, **82**:288-95.
2. Neidhardt FC: ***Escherichia coli* and *Salmonella typhimurium*: Cellular and molecular biology**. Edited by Neidhardt FC. Washington, D.C: American Society for Microbiology; 1987:4.
