# Supplementary figures and images for: Decrease of energy spilling in Escherichia coli continuous cultures with rising specific growth rate and carbon wasting
Source: BMC Syst Biol. 2011 Jul 5;5:106. doi: 10.1186/1752-0509-5-106 (PMC3149000; doi:10.1186/1752-0509-5-106)

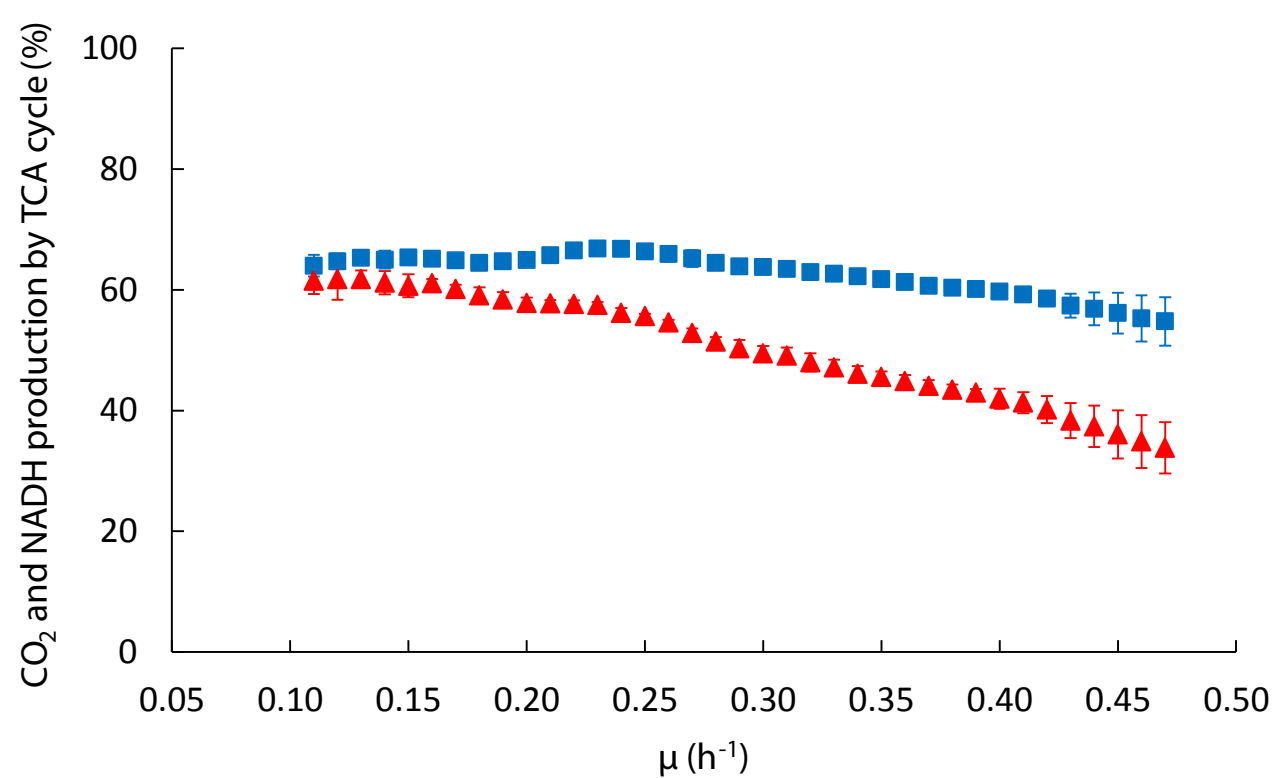

Supplement: Additional file 3 — E. coli K-12 MG1655 proportion of CO2 and NADH production by TCA cycle with rising specific growth rate in three A-stat cultivations. μ, specific growth rate (h-1). CO2 production (blue square); NADH production (red triangle). Error bars represent standard deviation of triplicate A-stat experiments. [file 1752-0509-5-106-S3.PDF]

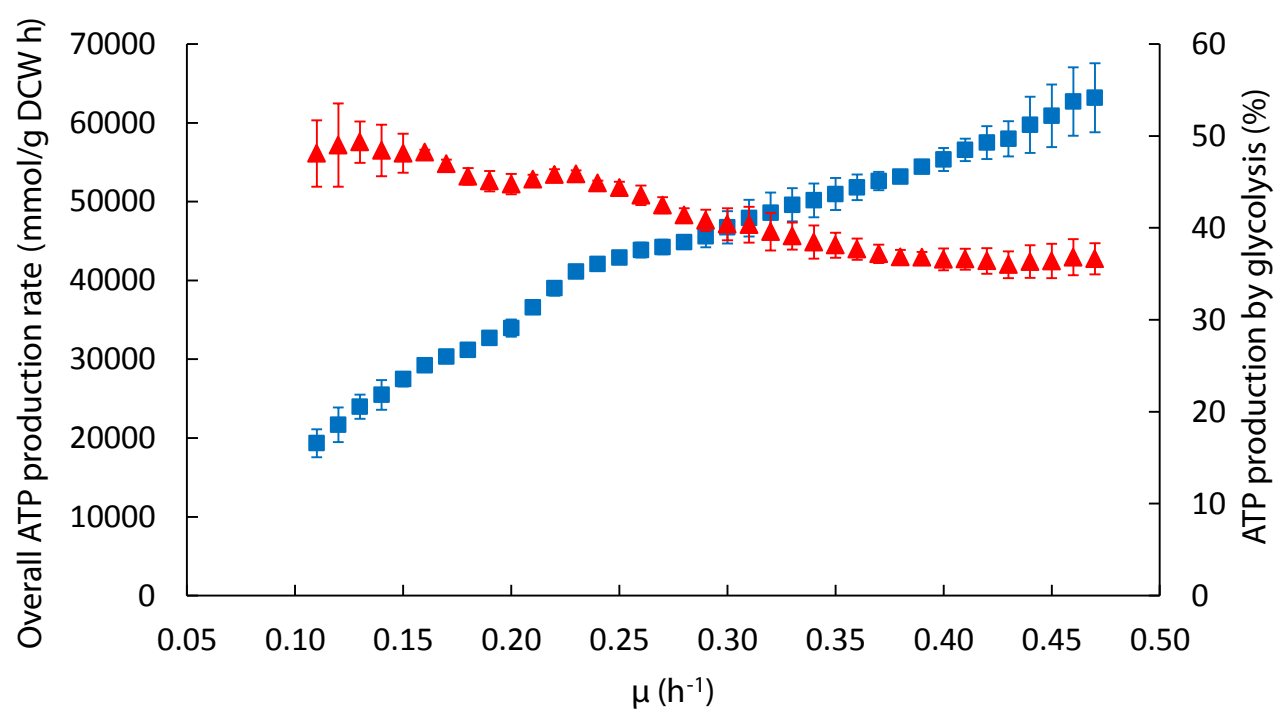

Supplement: Additional file 4 — Specific growth rate dependent overall ATP production rate and proportion of ATP production by glycolysis in three E. coli K-12 MG1655 A-stat cultivations. μ, specific growth rate (h-1). Overall ATP production (blue squares); ATP production by glycolysis (red triangle). Error bars represent standard deviation of triplicate A-stat experiments. [file 1752-0509-5-106-S4.PDF]

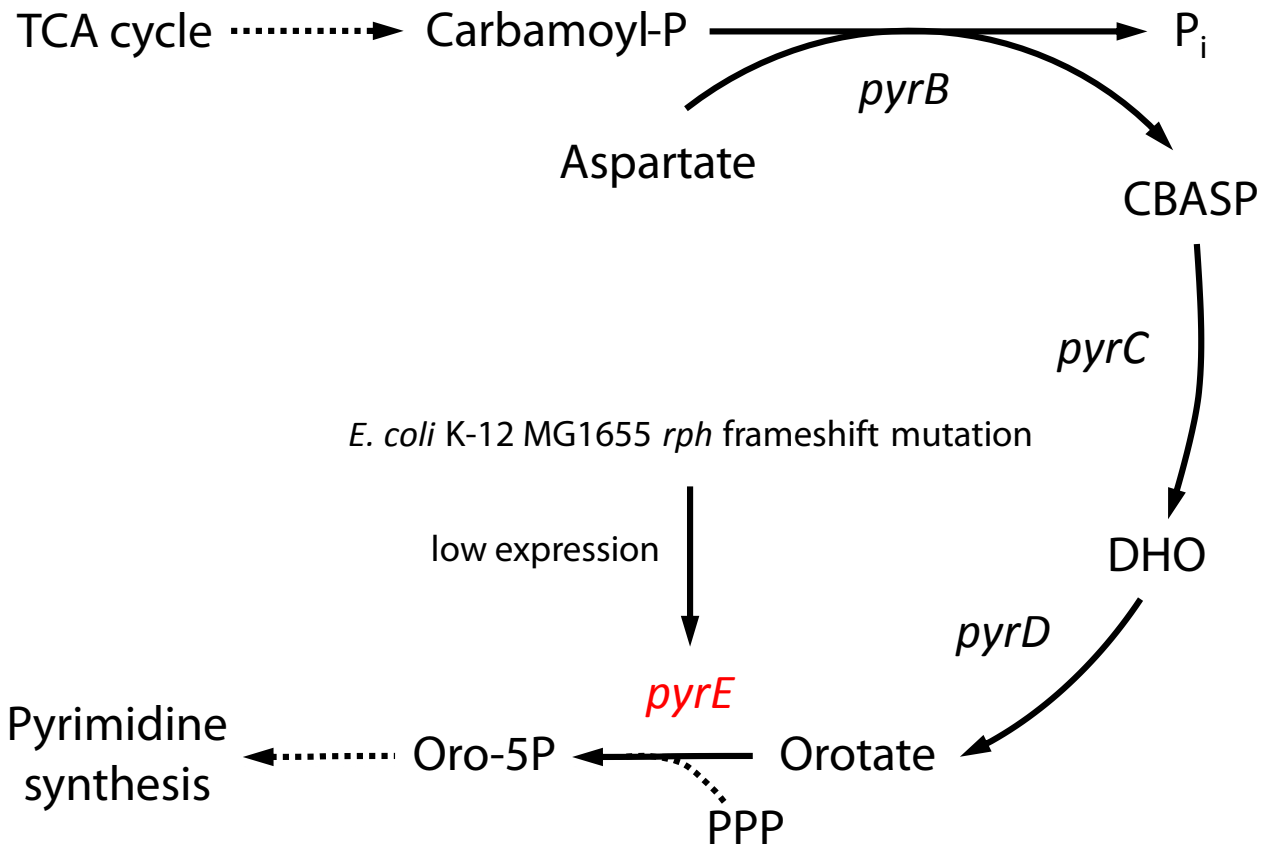

Supplement: Additional file 5 — E. coli K-12 MG1655 pyrimidine pathway rph frameshift mutation triggered accumulating precursor compounds. Carbamoyl-P, carbamoyl-phosphate; CBASP, carbamoyl-aspartate; DHO, dihydroorotate; Oro-5P, orotidine-5-phosphate; TCA cycle, tricarboxylic acid cycle; PPP, pentose phosphate pathway; pyrB, aspartate carbamoyltransferase; pyrC, dihydro-orotase; pyrD, dihydro-orotate oxidase; pyrE, orotate phosphoribosyltransferase. Gene names are italicised. [file 1752-0509-5-106-S5.PDF]
