# Supplementary material for: Decrease of energy spilling in Escherichia coli continuous cultures with rising specific growth rate and carbon wasting
Source: BMC Syst Biol. 2011 Jul 5;5:106. doi: 10.1186/1752-0509-5-106 (PMC3149000; doi:10.1186/1752-0509-5-106)
Supplement: Additional file 6 — Homology analysis for E. coli acetyltransferase prediction. BLAST results for homology analysis with Homo sapiens and Mus musculus aspartate N-acetyltransferases. [file 1752-0509-5-106-S6.PDF]

BLAST result for homology analysis with *Homo sapiens* aspartate N-acetyltransferase

BLASTP 2.2.25+
Reference: Stephen F. Altschul, Thomas L. Madden, Alejandro A. Schaffer, Jinghui Zhang, Zheng Zhang, Webb Miller, and David J. Lipman (1997), "Gapped BLAST and PSI-BLAST: a new generation of protein database search programs", Nucleic Acids Res. 25:3389-3402.

Reference for compositional score matrix adjustment: Stephen F. Altschul, John C. Wootton, E. Michael Gertz, Richa Agarwala, Aleksandr Morgulis, Alejandro A. Schaffer, and Yi-Kuo Yu (2005) "Protein database searches using compositionally adjusted substitution matrices", FEBS J. 272:5101-5109.

RID: TB9U2CT5014

Database: All non-redundant GenBank CDS translations+PDB+SwissProt+PIR+PRF excluding environmental samples from WGS projects
13,473,798 sequences; 4,621,495,809 total letters
Query= gi|119602947|gb|EAW82541.1| hypothetical protein LOC339983 [Homo sapiens]

Length=302

```
Score     E
Sequences producing significant alignments:
(Bits)  Value

ref|YP_026287.1|  predicted acetyltransferase [Escherichia col...
42.0    6e-05
gb|AAA97152.1|  ORF_f97 [Escherichia coli str. K-12 substr. MG...
40.4    2e-04
ref|NP_417898.1|  predicted acetyltransferase [Escherichia col...
38.9    6e-04
ref|AP_003028.1|  predicted acyltransferase with acyl-CoA N-ac...
33.5    0.028
ref|NP_418210.1|  predicted multidrug or homocysteine efflux s...
30.4    0.23
ref|NP_418790.1|  ribosomal-protein-S18-alanine N-acetyltransf...
30.0    0.26
gb|AAA97269.1|  CG Site No. 277 [Escherichia coli str. K-12 su...
30.0    0.28
```

ref|NP_417296.1| exonuclease V (RecBCD complex), alpha chain ...
30.0 0.30
ref|NP_417625.1| predicted acyltransferase with acyl-CoA N-ac...
28.9 0.56
ref|NP_415597.1| flagellar protein of basal-body outer-membra...
27.3 1.9
ref|NP_416506.1| CP4-44 prophage; predicted DNA repair protei...
26.2 3.7
ref|NP_418517.1| predicted acyltransferase with acyl-CoA N-ac...
25.4 6.3
ref|AP_002454.1| hypothetical protein [Escherichia coli str. ...
25.0 8.5
ref|NP_417059.1| pyridoxine 5'-phosphate synthase [Escherichi...
25.0 9.2

ALIGNMENTS
>ref|YP_026287.1| predicted acetyltransferase [Escherichia coli str.
K-12 substr.
MG1655]
ref|AP_004752.1| predicted acetyltransferase [Escherichia coli str.
K-12 substr.
W3110]
ref|YP_001733015.1| acetyltransferase [Escherichia coli str. K-12
substr. DH10B]
ref|YP_002929153.1| putative acetyltransferase [Escherichia coli
BW2952]
sp|P39337.2|YJGM_ECOLI RecName: Full=Uncharacterized N-
acetyltransferase YjgM
gb|AAT48245.1| predicted acetyltransferase [Escherichia coli str. K-
12 substr.
MG1655]
dbj|BAE78253.1| predicted acetyltransferase [Escherichia coli str.
K12 substr.
W3110]
gb|ACB05237.1| predicted acetyltransferase [Escherichia coli str. K-
12 substr.
DH10B]
gb|ACR64110.1| predicted acetyltransferase [Escherichia coli BW2952]
Length=167

Score = 42.0 bits (97), Expect = 6e-05, Method: Compositional matrix
adjust.
Identities = 36/102 (35%), Positives = 50/102 (49%), Gaps = 3/102
(3%)

```
Query  168  DMADIEQYYMKPPGSCFWVAVLDGNVVGI--
VAARAHEEDNTVELLRMSVDSRFRGKGIA  225
            ++ ++ Q Y +P G  +WV   +G VVG   +A     E +  EL +M
RGKG+A
Sbjct  47   NLDELYQVYSQP-
GHAYWVVEYEGEVVGGGGIAPLTGSESDICELQKMYFLPAIRGKGLA  105
```

```
Query  226  KALGRKVLEFAVVHNYSAVVLGTTAVKVAAHKLYESLGFRHM  267
            K L    +E A    +    L TTA    A  LYE LGF H+
Sbjct  106  KKLALMAMEQAREMGFKRCYLETTAFLKEAIALYEHLGFEHI  147


>gb|AAA97152.1| ORF_f97 [Escherichia coli str. K-12 substr. MG1655]
Length=97

 Score = 40.4 bits (93),  Expect = 2e-04, Method: Compositional matrix
adjust.
 Identities = 27/73 (37%), Positives = 34/73 (47%), Gaps = 0/73 (0%)

Query  195
GIVAARAHEEDNTVELLRMSVDSRFRGKGIAKALGRKVLEFAVVHNYSAVVLGTTAVKVA  254
            G +A     E +  EL +M      RGKG+AK L    +E A    +    L TTA
Sbjct  5
GGIAPLTGSESDICELQKMYFLPAIRGKGLAKKLALMAMEQAREMGFKRCYLETTAFLKE  64

Query  255  AHKLYESLGFRHM  267
            A  LYE LGF H+
Sbjct  65   AIALYEHLGFEHI  77


>ref|NP_417898.1| predicted acetyltransferase [Escherichia coli str.
K-12 substr.
MG1655]
 ref|AP_004351.1| predicted acetyltransferase [Escherichia coli str.
K-12 substr.
W3110]
 ref|YP_001732275.1| acetyltransferase [Escherichia coli str. K-12
substr. DH10B]
 ref|YP_002928328.1| putative acetyltransferase [Escherichia coli
BW2952]
 sp|P46854.1|YHHY_ECOLI RecName: Full=Uncharacterized N-
acetyltransferase YhhY
 gb|AAA58239.1| ORF_o162 [Escherichia coli str. K-12 substr. MG1655]
 gb|AAC76466.1| predicted acetyltransferase [Escherichia coli str. K-
12 substr.
MG1655]
 dbj|BAE77852.1| predicted acetyltransferase [Escherichia coli str.
K12 substr.
W3110]
 gb|ACB04497.1| predicted acetyltransferase [Escherichia coli str. K-
12 substr.
DH10B]
 gb|ACR64111.1| predicted acetyltransferase [Escherichia coli BW2952]
Length=162

 Score = 38.9 bits (89),  Expect = 6e-04, Method: Compositional matrix
adjust.
 Identities = 30/101 (30%), Positives = 47/101 (47%), Gaps = 7/101
(7%)
```

```
Query  180  PGSCFWVAVLDGNVVG--
IVAARAHEEDNTVELLRMSVDSRFRGKGIAKALGRKVLEFAV  237
            PG    VA +DG+VVG   +  +     + V    + VDSR++ +G+A AL R+++E
Sbjct  49
PGIKQLVACIDGDVVGHLTIDVQQRPRRSHVADFGICVDSRWKNRGVASALMREMIEMC-  107

Query  238  VHNY---SAVVLGTTAVKVAAHKLYESLGFRHMGASDHYVL  275
              N+     + L        A K+Y+  GF   G    Y L
Sbjct  108  -DNWLRVDRIELTVFVDNAPAIKVYKKYGFEIEGTGKKYAL  147
```

```
>ref|AP_003028.1| predicted acyltransferase with acyl-CoA N-
acyltransferase domain
[Escherichia coli str. K-12 substr. W3110]
 ref|NP_416929.4| predicted acyltransferase with acyl-CoA N-
acyltransferase domain
[Escherichia coli str. K-12 substr. MG1655]
 ref|YP_001731363.1| acyltransferase [Escherichia coli str. K-12
substr. DH10B]
 ref|YP_002927393.1| putative acyltransferase with acyl-CoA N-
acyltransferase domain
[Escherichia coli BW2952]
 sp|P76539.2|YPEA_ECOLI RecName: Full=Acetyltransferase YpeA
 dbj|BAE76714.1| predicted acyltransferase with acyl-CoA N-
acyltransferase domain
[Escherichia coli str. K12 substr. W3110]
 gb|AAC75487.2| predicted acyltransferase with acyl-CoA N-
acyltransferase domain
[Escherichia coli str. K-12 substr. MG1655]
 gb|ACB03585.1| predicted acyltransferase with acyl-CoA N-
acyltransferase domain
[Escherichia coli str. K-12 substr. DH10B]
 gb|ACR64125.1| predicted acyltransferase with acyl-CoA N-
acyltransferase domain
[Escherichia coli BW2952]
Length=141

 Score = 33.5 bits (75),  Expect = 0.028, Method: Compositional matrix
adjust.
 Identities = 34/122 (28%), Positives = 49/122 (40%), Gaps = 13/122
(11%)

Query  171
DIEQYYMKPPGSCFWVAVLDGNVVGIVAARAHEEDNTVELLRMSVDSRFRGKGIAKALGR  230
            DIE+   M    S F VA ++G+VVG V          +    L   V   FRG+GIA AL
Sbjct  33   DIERK-MNHDVSLFLVAEVNGDVVGTVMGGYDGHRGSAYYL--
GVHPEFRGRGIANALLN  89

Query  231
KVLEFAVVHNYSAVVLGTTAVKVAAHKLYESLGFRHMGASDHYVLPGMTLSLAERLFFQV  290
            ++ +  +      + +           +YE LG+ H             LSL +RL
```

```
Sbjct  90   RLEKKLIARGCPKIQINVPEDNDMVLGMYERLGYEHAD----------
VLSLGKRLIEDE  139

Query  291  RY  292
             Y
Sbjct  140  EY  141


>ref|NP_418210.1| predicted multidrug or homocysteine efflux system
[Escherichia
coli str. K-12 substr. MG1655]
 ref|AP_004033.1| predicted multidrug or homocysteine efflux system
[Escherichia
coli str. K-12 substr. W3110]
 ref|YP_001732573.1| multidrug or homocysteine efflux system
[Escherichia coli str.
K-12 substr. DH10B]
 ref|YP_002928642.1| putative multidrug or homocysteine efflux system
[Escherichia
coli BW2952]
 sp|P31474.1|HSRA_ECOLI RecName: Full=Probable transport protein hsrA;
AltName: Full=High-copy
suppressor of rspA
 gb|AAC76777.1| predicted multidrug or homocysteine efflux system
[Escherichia
coli str. K-12 substr. MG1655]
 dbj|BAE77534.1| predicted multidrug or homocysteine efflux system
[Escherichia
coli str. K12 substr. W3110]
 gb|ACB04795.1| predicted multidrug or homocysteine efflux system
[Escherichia
coli str. K-12 substr. DH10B]
 gb|ACR64683.1| predicted multidrug or homocysteine efflux system
[Escherichia
coli BW2952]
Length=475

 Score = 30.4 bits (67),  Expect = 0.23, Method: Compositional matrix
adjust.
 Identities = 23/111 (21%), Positives = 48/111 (43%), Gaps = 10/111
(9%)

Query  172
IEQYYMKPPGSCFWVAVLDGNVVGIVAARAHEEDNTVELLRMSVDSRFRGKGIAKALGRK  231
            I Q+ ++ P    W+ +L   ++G+  +      NT+ L  ++ D+   G  +
+
Sbjct  342
IAQFSLQSPAMAIWMLILPLFILGMAMSTQFTAMNTITLADLTDDNASSGNSVLAVTQQL  401

Query  232  VLEFAVVHNYSAVVL-------GTTAVKVAAHKLYESLGFRHMGASDHYVL  275
             +   V    SA VL       GTT V+   H  + ++G   + ++  ++L
Sbjct  402  SISLGVA--VSAAVLRVYEGMEGTTTVE-QFHYTFITMGIITVASAAMFML  449
```

```
>ref|NP_418790.1| ribosomal-protein-S18-alanine N-acetyltransferase
[Escherichia
coli str. K-12 substr. MG1655]
 ref|AP_004860.1| acetylase for 30S ribosomal subunit protein S18
[Escherichia
coli str. K-12 substr. W3110]
 ref|YP_001733078.1| acetylase for 30S ribosomal subunit protein S18
[Escherichia
coli str. K-12 substr. DH10B]
 ref|YP_002929255.1| acetylase for 30S ribosomal subunit protein S18
[Escherichia
coli BW2952]
 sp|P0A944.1|RIMI_ECOLI RecName: Full=Ribosomal-protein-alanine
acetyltransferase; AltName:
Full=Acetylating enzyme for N-terminal of ribosomal protein
S18
 gb|AAC77326.1| ribosomal-protein-S18-alanine N-acetyltransferase
[Escherichia
coli str. K-12 substr. MG1655]
 dbj|BAE78361.1| acetylase for 30S ribosomal subunit protein S18
[Escherichia
coli str. K12 substr. W3110]
 gb|ACB05300.1| acetylase for 30S ribosomal subunit protein S18
[Escherichia
coli str. K-12 substr. DH10B]
 gb|ACR61906.1| acetylase for 30S ribosomal subunit protein S18
[Escherichia
coli BW2952]
Length=148

 Score = 30.0 bits (66),  Expect = 0.26, Method: Compositional matrix
adjust.
 Identities = 18/64 (28%), Positives = 31/64 (48%), Gaps = 0/64 (0%)

Query  210
LLRMSVDSRFRGKGIAKALGRKVLEFAVVHNYSAVVLGTTAVKVAAHKLYESLGFRHMGA  269
            L  ++VD  ++ +G+ +AL   +++       + + L   A   AA  LYESLGF
Sbjct  66
LFNIAVDPDYQRQGLGRALLEHLIDELEKRGVATLWLEVRASNAAAIALYESLGFNEATI  125

Query  270  SDHY  273
              +Y
Sbjct  126  RRNY  129


>gb|AAA97269.1| CG Site No. 277 [Escherichia coli str. K-12 substr.
MG1655]
Length=161
```

```
 Score = 30.0 bits (66),  Expect = 0.28, Method: Compositional matrix
adjust.
 Identities = 18/64 (28%), Positives = 31/64 (48%), Gaps = 0/64 (0%)

Query  210
LLRMSVDSRFRGKGIAKALGRKVLEFAVVHNYSAVVLGTTAVKVAAHKLYESLGFRHMGA  269
            L  ++VD  ++ +G+ +AL   +++       + + L   A   AA  LYESLGF
Sbjct  66
LFNIAVDPDYQRQGLGRALLEHLIDELEKRGVATLWLEVRASNAAAIALYESLGFNEATI  125

Query  270  SDHY  273
              +Y
Sbjct  126  RRNY  129


>ref|NP_417296.1| exonuclease V (RecBCD complex), alpha chain
[Escherichia coli
str. K-12 substr. MG1655]
 ref|AP_003382.1| exonuclease V (RecBCD complex), alpha chain
[Escherichia coli
str. K-12 substr. W3110]
 ref|YP_001731707.1| exonuclease V (RecBCD complex), alpha chain
[Escherichia coli
str. K-12 substr. DH10B]
 ref|YP_002927750.1| exonuclease V (RecBCD complex), alpha chain
[Escherichia coli
BW2952]
 sp|P04993.2|EX5A_ECOLI RecName: Full=Exodeoxyribonuclease V alpha
chain; AltName: Full=Exodeoxyribonuclease
V 67 kDa polypeptide
 pdb|3K70|D Chain D, Crystal Structure Of The Complete Initiation
Complex
Of Recbcd
 pdb|3K70|G Chain G, Crystal Structure Of The Complete Initiation
Complex
Of Recbcd
 gb|AAB40466.1| exonuclease V alpha-subunit [Escherichia coli str. K-
12 substr.
MG1655]
 gb|AAC75858.1| exonuclease V (RecBCD complex), alpha chain
[Escherichia coli
str. K-12 substr. MG1655]
 dbj|BAE76888.1| exonuclease V (RecBCD complex), alpha chain
[Escherichia coli
str. K12 substr. W3110]
 gb|ACB03929.1| exonuclease V (RecBCD complex), alpha chain
[Escherichia coli
str. K-12 substr. DH10B]
 gb|ACR63050.1| exonuclease V (RecBCD complex), alpha chain
[Escherichia coli
BW2952]
Length=608
```

Score = 30.0 bits (66), Expect = 0.30, Method: Compositional matrix adjust.
Identities = 18/59 (31%), Positives = 28/59 (47%), Gaps = 0/59 (0%)

```
Query  149
RYYYSRKVIRAYLECALHTDMADIEQYYMKPPGSCFWVAVLDGNVVGIVAARAHEEDNT  207
           R+Y  R V+ A  + AL     DI     +  G+  W A+ DGN+  +  +R  E +
T
Sbjct  475
RWYEGRPVMIARNDSALGLFNGDIGIALDRGQGTRVWFAMPDGNIKSVQPSRLPEHETT  533
```

>ref|NP_417625.1| predicted acyltransferase with acyl-CoA N-acyltransferase domain
[Escherichia coli str. K-12 substr. MG1655]
ref|AP_003701.1| predicted acyltransferase with acyl-CoA N-acyltransferase domain
[Escherichia coli str. K-12 substr. W3110]
ref|YP_001732013.1| acyltransferase [Escherichia coli str. K-12 substr. DH10B]
ref|YP_002928056.1| putative acyltransferase with acyl-CoA N-acyltransferase domain
[Escherichia coli BW2952]
sp|P63417.1|YHBS_ECOLI RecName: Full=Uncharacterized N-acetyltransferase YhbS
gb|AAA57959.1| ORF_f167; end overlaps end of o100 by 14 bases; start overlaps
f174, other starts possible [Escherichia coli str. K-12 substr. MG1655]
gb|AAC76190.1| predicted acyltransferase with acyl-CoA N-acyltransferase domain
[Escherichia coli str. K-12 substr. MG1655]
dbj|BAE77202.1| predicted acyltransferase with acyl-CoA N-acyltransferase domain
[Escherichia coli str. K12 substr. W3110]
gb|ACB04235.1| predicted acyltransferase with acyl-CoA N-acyltransferase domain
[Escherichia coli str. K-12 substr. DH10B]
gb|ACR64126.1| predicted acyltransferase with acyl-CoA N-acyltransferase domain
[Escherichia coli BW2952]
Length=167

Score = 28.9 bits (63), Expect = 0.56, Method: Compositional matrix adjust.
Identities = 27/81 (33%), Positives = 40/81 (49%), Gaps = 12/81 (15%)

```
Query  190  DGNVVGIVA---ARAHEED-
NTVELLRMSVDSRFRGKGIAKALGRKVLEFAVVHNYSAVV  245
            +G V+G VA        ED   V +  ++VD ++RG+G+A+ L  + L+
Y+AVV
```

```
Sbjct  50
EGQVIGYVAFSPVDVQGEDLQWVGMAPLAVDEKYRGQGLARQLVYEGLDSLNEFGYAAVV  109

Query  246  -LGTTAVKVAAHKLYESLGFR  265
             LG  A       LY   GF
Sbjct  110  TLGDPA-------LYSRFGFE  123
```

>ref|NP_415597.1| flagellar protein of basal-body outer-membrane L ring [Escherichia
coli str. K-12 substr. MG1655]
 ref|AP_001705.1| flagellar protein of basal-body outer-membrane L ring [Escherichia
coli str. K-12 substr. W3110]
 ref|YP_001730050.1| flagellar protein of basal-body outer-membrane L ring [Escherichia
coli str. K-12 substr. DH10B]
 ref|YP_002926129.1| flagellar protein of basal-body outer-membrane L ring [Escherichia
coli BW2952]
 sp|P0A6S0.1|FLGH_ECOLI RecName: Full=Flagellar L-ring protein; AltName: Full=Basal body
L-ring protein; Flags: Precursor
 sp|B1X9J2.1|FLGH_ECODH RecName: Full=Flagellar L-ring protein; AltName: Full=Basal body
L-ring protein; Flags: Precursor
 sp|C4ZS19.1|FLGH_ECOBW RecName: Full=Flagellar L-ring protein; AltName: Full=Basal body
L-ring protein; Flags: Precursor
 gb|AAC74163.1| flagellar protein of basal-body outer-membrane L ring [Escherichia
coli str. K-12 substr. MG1655]
 dbj|BAA35888.2| flagellar protein of basal-body outer-membrane L ring [Escherichia
coli str. K12 substr. W3110]
 gb|ACB02272.1| flagellar protein of basal-body outer-membrane L ring [Escherichia
coli str. K-12 substr. DH10B]
 gb|ACR63114.1| flagellar protein of basal-body outer-membrane L ring [Escherichia
coli BW2952]
Length=232

 Score = 27.3 bits (59), Expect = 1.9, Method: Compositional matrix adjust.
 Identities = 13/33 (39%), Positives = 16/33 (48%), Gaps = 1/33 (3%)

```
Query  31  LLAAAGAMW-PPLPAAPGPAAAPPAPPPAPVAQ  62
           +L+   G   W P   P    G   +A  P  P  P PVA
Sbjct  16  VLSLTGCAWIPSTPLVQGATSAQPVPGPTPVAN  48
```

```
>ref|NP_416506.1| CP4-44 prophage; predicted DNA repair protein
[Escherichia coli
str. K-12 substr. MG1655]
 ref|AP_002601.1| predicted DNA repair protein [Escherichia coli str.
K-12 substr.
W3110]
 ref|YP_001730951.1| CP4-44 prophage; DNA repair protein [Escherichia
coli str. K-12
substr. DH10B]
 ref|YP_002926989.1| CP4-44 prophage; putative DNA repair protein
[Escherichia coli
BW2952]
 sp|P76362.1|YEES_ECOLI RecName: Full=UPF0758 protein yeeS
 dbj|BAA15826.1| predicted DNA repair protein [Escherichia coli str.
K12 substr.
W3110]
 gb|AAC75063.1| CP4-44 prophage; predicted DNA repair protein
[Escherichia coli
str. K-12 substr. MG1655]
 gb|ACB03173.1| CP4-44 prophage; predicted DNA repair protein
[Escherichia coli
str. K-12 substr. DH10B]
 gb|ACR62614.1| CP4-44 prophage; predicted DNA repair protein
[Escherichia coli
BW2952]
Length=148

 Score = 26.2 bits (56),  Expect = 3.7, Method: Compositional matrix
adjust.
 Identities = 19/66 (29%), Positives = 33/66 (50%), Gaps = 11/66 (17%)

Query  230  RKVLEFAVVHNYSAVVLG----------
TTAVKVAAHKLYESLGFRHMGASDHYVLPG-M  278
            R+V++ A+ HN +AVVL           + A ++   +L ++LG   +   DH ++ G
Sbjct  79
REVIKRALYHNAAAVVLAHNHPSGEVTPSKADRLITERLVQALGLVDIRVPDHLIVGGNQ  138

Query  279  TLSLAE  284
              S AE
Sbjct  139  VFSFAE  144


>ref|NP_418517.1| predicted acyltransferase with acyl-CoA N-
acyltransferase domain
[Escherichia coli str. K-12 substr. MG1655]
 ref|AP_004595.1| predicted acyltransferase with acyl-CoA N-
acyltransferase domain
[Escherichia coli str. K-12 substr. W3110]
 ref|YP_001732867.1| acyltransferase [Escherichia coli str. K-12
substr. DH10B]
 ref|YP_002928999.1| putative acyltransferase with acyl-CoA N-
acyltransferase domain
```

[Escherichia coli BW2952]
 sp|P16691.1|PHNO_ECOLI RecName: Full=Protein PhnO
 dbj|BAA14275.1| phnO [Escherichia coli W3110]
 gb|AAA96992.1| phnO [Escherichia coli str. K-12 substr. MG1655]
 gb|AAC77054.1| predicted acyltransferase with acyl-CoA N-acyltransferase domain
[Escherichia coli str. K-12 substr. MG1655]
 dbj|BAE78096.1| predicted acyltransferase with acyl-CoA N-acyltransferase domain
[Escherichia coli str. K12 substr. W3110]
 gb|ACB05089.1| predicted acyltransferase with acyl-CoA N-acyltransferase domain
[Escherichia coli str. K-12 substr. DH10B]
 gb|ACR64127.1| predicted acyltransferase with acyl-CoA N-acyltransferase domain
[Escherichia coli BW2952]
Length=144

```
 Score = 25.4 bits (54),  Expect = 6.3, Method: Compositional matrix adjust.
 Identities = 22/92 (24%), Positives = 37/92 (40%), Gaps = 4/92 (4%)

Query  177  MKPPGSCFWVAVLDGNVVGIVAA----RAHEEDNTVELLRMSVDSRFRGKGIAKALGRKV  232
            ++ P   + +A+LDG VVG++        H  +   E+  + V  + RG  +   L
Sbjct  42   LRDPNMRYHLALLDGEVVGMIGLHLQFHLHHVNWIGEIQELVVMPQARGLNVGSKLLAWA  101

Query  233  LEFAVVHNYSAVVLGTTAVKVAAHKLYESLGF  264
             E A         L T   +  AH+ Y   G+
Sbjct  102  EEEARQAGAEMTELSTNVKRHDAHRFYLREGY  133
```

>ref|AP_002454.1| hypothetical protein [Escherichia coli str. K-12 substr. W3110]
 ref|NP_416348.2| conserved protein [Escherichia coli str. K-12 substr. MG1655]
 ref|YP_001730810.1| hypothetical protein ECDH10B_1973 [Escherichia coli str. K-12
substr. DH10B]
 ref|YP_002926845.1| hypothetical protein BWG_1648 [Escherichia coli BW2952]
 sp|P76272.2|YEBT_ECOLI RecName: Full=Uncharacterized protein yebT
 dbj|BAA15647.1| conserved hypothetical protein [Escherichia coli str. K12 substr.
W3110]
 gb|AAC74904.2| conserved protein [Escherichia coli str. K-12 substr. MG1655]
 gb|ACB03032.1| conserved protein [Escherichia coli str. K-12 substr. DH10B]
 gb|ACR62492.1| conserved protein [Escherichia coli BW2952]
Length=877

```
 Score = 25.0 bits (53),  Expect = 8.5, Method: Compositional matrix
adjust.
 Identities = 16/67 (24%), Positives = 33/67 (49%), Gaps = 9/67 (13%)

Query  153  SRKVIRAYLECALHTDMAD-----IEQYYMKPPGSCFWVAVLD----
GNVVGIVAARAHE  203
            S  + +  ++ ++ +DM D      + + + P  S   V+ LD    GN +G++  +
E
Sbjct  82
SDDLRKIEVKVSIKSDMKDALREETQFWLVTPKASLAGVSGLDALVGGNYIGMMPGKGKE  141

Query  204  EDNTVEL  210
            +D+ V L
Sbjct  142  QDHFVAL  148


>ref|NP_417059.1| pyridoxine 5'-phosphate synthase [Escherichia coli
str. K-12
substr. MG1655]
 ref|AP_003150.1| pyridoxine 5'-phosphate synthase [Escherichia coli
str. K-12
substr. W3110]
 ref|YP_001731493.1| pyridoxine 5'-phosphate synthase [Escherichia
coli str. K-12
substr. DH10B]
 ref|YP_002927525.1| pyridoxine 5'-phosphate synthase [Escherichia
coli BW2952]
 sp|P0A794.2|PDXJ_ECOLI RecName: Full=Pyridoxine 5'-phosphate
synthase; Short=PNP synthase
 gb|AAA79826.1| CG Site No. 416 [Escherichia coli str. K-12 substr.
MG1655]
 gb|AAC75617.1| pyridoxine 5'-phosphate synthase [Escherichia coli
str. K-12
substr. MG1655]
 dbj|BAE76740.1| pyridoxine 5'-phosphate synthase [Escherichia coli
str. K12 substr.
W3110]
 gb|ACB03715.1| pyridoxine 5'-phosphate synthase [Escherichia coli
str. K-12
substr. DH10B]
 gb|ACR65350.1| pyridoxine 5'-phosphate synthase [Escherichia coli
BW2952]
Length=243

 Score = 25.0 bits (53),  Expect = 9.2, Method: Compositional matrix
adjust.
 Identities = 17/59 (29%), Positives = 24/59 (41%), Gaps = 12/59 (20%)

Query  190
DGNVVGIVAARAHEEDNTVELLRMSVDSRFRGKGIAKALGRKVLEFAVVHNYSAVVLGT  248
```

```
            DG  V +   R H  D  V +LR ++D+R              LE AV     A+ +
T
Sbjct  40   DGITVHLREDRRHITDRDVRILRQTLDTRMN------------
LEMAVTEEMLAIAVET  86
```

```
  Database: All non-redundant GenBank CDS
translations+PDB+SwissProt+PIR+PRF
excluding environmental samples from WGS projects
    Posted date:  Mar 22, 2011  4:36 PM
  Number of letters in database: 1,896,201
  Number of sequences in database:  6,138

Lambda     K      H
   0.324    0.138    0.428
Gapped
Lambda     K      H
   0.267   0.0410    0.140
Matrix: BLOSUM62
Gap Penalties: Existence: 11, Extension: 1
Number of Sequences: 6138
Number of Hits to DB: 919961
Number of extensions: 38677
Number of successful extensions: 168
Number of sequences better than 100: 73
Number of HSP's better than 100 without gapping: 0
Number of HSP's gapped: 164
Number of HSP's successfully gapped: 81
Length of query: 302
Length of database: 1896201
Length adjustment: 86
Effective length of query: 216
Effective length of database: 1368333
Effective search space: 295559928
Effective search space used: 295559928
T: 11
A: 40
X1: 15 (7.0 bits)
X2: 38 (14.6 bits)
X3: 64 (24.7 bits)
S1: 40 (20.0 bits)
S2: 44 (21.6 bits)
```

BLAST result for homology analysis with *Mus musculus* aspartate N-acetyltransferase

BLASTP 2.2.25+
Reference: Stephen F. Altschul, Thomas L. Madden, Alejandro A. Schaffer, Jinghui Zhang, Zheng Zhang, Webb Miller, and David J. Lipman (1997), "Gapped BLAST and PSI-BLAST: a new generation of protein database search programs", Nucleic Acids Res. 25:3389-3402.

Reference for compositional score matrix adjustment: Stephen F. Altschul, John C. Wootton, E. Michael Gertz, Richa Agarwala, Aleksandr Morgulis, Alejandro A. Schaffer, and Yi-Kuo Yu (2005) "Protein database searches using compositionally adjusted substitution matrices", FEBS J. 272:5101-5109.

RID: TB9YZDB401S

Database: All non-redundant GenBank CDS translations+PDB+SwissProt+PIR+PRF excluding environmental samples from WGS projects
13,473,798 sequences; 4,621,495,809 total letters
Query= gi|134288912|ref|NP_001001985.3| N-acetyltransferase 8-like protein
[Mus musculus]

Length=299

```
Score     E
Sequences producing significant alignments:
(Bits)  Value

ref|YP_026287.1|  predicted acetyltransferase [Escherichia col...
42.4    6e-05
ref|NP_417898.1|  predicted acetyltransferase [Escherichia col...
40.4    2e-04
gb|AAA97152.1|  ORF_f97 [Escherichia coli str. K-12 substr. MG...
40.4    2e-04
ref|AP_003028.1|  predicted acyltransferase with acyl-CoA N-ac...
33.9    0.020
ref|NP_417296.1|  exonuclease V (RecBCD complex), alpha chain ...
32.3    0.062
gb|AAA97269.1|  CG Site No. 277 [Escherichia coli str. K-12 su...
30.4    0.22
ref|NP_418790.1|  ribosomal-protein-S18-alanine N-acetyltransf...
30.0    0.25
```

ref|NP_418210.1| predicted multidrug or homocysteine efflux s...
29.3   0.43
ref|NP_417625.1| predicted acyltransferase with acyl-CoA N-ac...
28.9   0.58
ref|NP_417454.1| DNA-binding transcriptional dual regulator, ...
27.3   1.8
gb|AAA69147.1| ORF_o274 [Escherichia coli str. K-12 substr. M...
27.3   2.0
ref|YP_001730194.1| ATP-binding subunit of oligopeptide ABC t...
26.6   2.8
ref|NP_415763.1| oligopeptide transporter subunit [Escherichi...
26.6   2.8
ref|NP_416506.1| CP4-44 prophage; predicted DNA repair protei...
26.6   2.9
ref|NP_418517.1| predicted acyltransferase with acyl-CoA N-ac...
25.8   5.4
ref|AP_004342.1| glycerol-3-phosphate transporter subunit [Es...
25.0   7.7
ref|AP_002454.1| hypothetical protein [Escherichia coli str. ...
25.0   7.8
gb|AAB18425.1| CG Site No. 17794 [Escherichia coli str. K-12 ...
25.0   7.9
ref|NP_417259.1| enolase [Escherichia coli str. K-12 substr. ...
25.0   9.2
ref|NP_418334.1| predicted peptidase [Escherichia coli str. K...
25.0   9.4
ref|NP_416307.1| conserved outer membrane lipoprotein [Escher...
25.0   9.7

ALIGNMENTS
>ref|YP_026287.1| predicted acetyltransferase [Escherichia coli str. K-12 substr.
MG1655]
ref|AP_004752.1| predicted acetyltransferase [Escherichia coli str. K-12 substr.
W3110]
ref|YP_001733015.1| acetyltransferase [Escherichia coli str. K-12 substr. DH10B]
ref|YP_002929153.1| putative acetyltransferase [Escherichia coli BW2952]
sp|P39337.2|YJGM_ECOLI RecName: Full=Uncharacterized N-acetyltransferase YjgM
gb|AAT48245.1| predicted acetyltransferase [Escherichia coli str. K-12 substr.
MG1655]
dbj|BAE78253.1| predicted acetyltransferase [Escherichia coli str. K12 substr.
W3110]
gb|ACB05237.1| predicted acetyltransferase [Escherichia coli str. K-12 substr.
DH10B]
gb|ACR64110.1| predicted acetyltransferase [Escherichia coli BW2952]

Length=167

 Score = 42.4 bits (98), Expect = 6e-05, Method: Compositional matrix adjust.
 Identities = 36/102 (35%), Positives = 50/102 (49%), Gaps = 3/102 (3%)

```
Query  165  DMADIEQYYMKPPGSCFWVAVLDGNVVGI--
VAARAHEEDNTVELLRMSVDSRFRGKGIA  222
            ++ ++ Q Y +P G  +WV   +G VVG   +A     E +  EL +M
RGKG+A
Sbjct  47   NLDELYQVYSQP-
GHAYWVVEYEGEVVGGGGIAPLTGSESDICELQKMYFLPAIRGKGLA  105

Query  223  KALGRRVLEFAMLHNYSAVVLGTTAVKVAAHKLYESLGFRHM  264
            K L    +E A    +    L TTA    A  LYE LGF H+
Sbjct  106  KKLALMAMEQAREMGFKRCYLETTAFLKEAIALYEHLGFEHI  147
```

>ref|NP_417898.1| predicted acetyltransferase [Escherichia coli str. K-12 substr.
MG1655]
 ref|AP_004351.1| predicted acetyltransferase [Escherichia coli str. K-12 substr.
W3110]
 ref|YP_001732275.1| acetyltransferase [Escherichia coli str. K-12 substr. DH10B]
 ref|YP_002928328.1| putative acetyltransferase [Escherichia coli BW2952]
 sp|P46854.1|YHHY_ECOLI RecName: Full=Uncharacterized N-acetyltransferase YhhY
 gb|AAA58239.1| ORF_o162 [Escherichia coli str. K-12 substr. MG1655]
 gb|AAC76466.1| predicted acetyltransferase [Escherichia coli str. K-12 substr.
MG1655]
 dbj|BAE77852.1| predicted acetyltransferase [Escherichia coli str. K12 substr.
W3110]
 gb|ACB04497.1| predicted acetyltransferase [Escherichia coli str. K-12 substr.
DH10B]
 gb|ACR64111.1| predicted acetyltransferase [Escherichia coli BW2952]
Length=162

 Score = 40.4 bits (93), Expect = 2e-04, Method: Compositional matrix adjust.
 Identities = 31/101 (31%), Positives = 47/101 (47%), Gaps = 7/101 (7%)

```
Query  177  PGSCFWVAVLDGNVVG--
IVAARAHEEDNTVELLRMSVDSRFRGKGIAKALGRRVLEFAM  234
```

```
           PG    VA +DG+VVG   +  +     + V    + VDSR++ +G+A AL R ++E
M
Sbjct  49   PGIKQLVACIDGDVVGHLTIDVQQRPRRSHVADFGICVDSRWKNRGVASALMREMIE-
-M  106

Query  235  LHNY---SAVVLGTTAVKVAAHKLYESLGFRHMGASDHYVL  272
              N+     + L        A K+Y+   GF   G    Y L
Sbjct  107  CDNWLRVDRIELTVFVDNAPAIKVYKKYGFEIEGTGKKYAL  147


>gb|AAA97152.1| ORF_f97 [Escherichia coli str. K-12 substr. MG1655]
Length=97

 Score = 40.4 bits (93),  Expect = 2e-04, Method: Compositional matrix
adjust.
 Identities = 27/73 (37%), Positives = 34/73 (47%), Gaps = 0/73 (0%)

Query  192
GIVAARAHEEDNTVELLRMSVDSRFRGKGIAKALGRRVLEFAMLHNYSAVVLGTTAVKVA  251
            G +A     E +  EL +M      RGKG+AK L    +E A    +    L TTA
Sbjct  5
GGIAPLTGSESDICELQKMYFLPAIRGKGLAKKLALMAMEQAREMGFKRCYLETTAFLKE  64

Query  252  AHKLYESLGFRHM  264
            A  LYE LGF H+
Sbjct  65   AIALYEHLGFEHI  77


>ref|AP_003028.1| predicted acyltransferase with acyl-CoA N-
acyltransferase domain
[Escherichia coli str. K-12 substr. W3110]
 ref|NP_416929.4| predicted acyltransferase with acyl-CoA N-
acyltransferase domain
[Escherichia coli str. K-12 substr. MG1655]
 ref|YP_001731363.1| acyltransferase [Escherichia coli str. K-12
substr. DH10B]
 ref|YP_002927393.1| putative acyltransferase with acyl-CoA N-
acyltransferase domain
[Escherichia coli BW2952]
 sp|P76539.2|YPEA_ECOLI RecName: Full=Acetyltransferase YpeA
 dbj|BAE76714.1| predicted acyltransferase with acyl-CoA N-
acyltransferase domain
[Escherichia coli str. K12 substr. W3110]
 gb|AAC75487.2| predicted acyltransferase with acyl-CoA N-
acyltransferase domain
[Escherichia coli str. K-12 substr. MG1655]
 gb|ACB03585.1| predicted acyltransferase with acyl-CoA N-
acyltransferase domain
[Escherichia coli str. K-12 substr. DH10B]
 gb|ACR64125.1| predicted acyltransferase with acyl-CoA N-
acyltransferase domain
[Escherichia coli BW2952]
```

Length=141

 Score = 33.9 bits (76), Expect = 0.020, Method: Compositional matrix
adjust.
 Identities = 35/122 (29%), Positives = 49/122 (40%), Gaps = 13/122
(11%)

```
Query  168
DIEQYYMKPPGSCFWVAVLDGNVVGIVAARAHEEDNTVELLRMSVDSRFRGKGIAKALGR  227
           DIE+  M    S F VA ++G+VVG V          +   L   V   FRG+GIA AL
Sbjct  33   DIERK-MNHDVSLFLVAEVNGDVVGTVMGGYDGHRGSAYYL--
GVHPEFRGRGIANALLN  89

Query  228
RVLEFAMLHNYSAVVLGTTAVKVAAHKLYESLGFRHMGASDHYVLPGMTLSLAERLFFQV  287
           R+ +  +      + +           +YE LG+ H              LSL +RL
Sbjct  90   RLEKKLIARGCPKIQINVPEDNDMVLGMYERLGYEHAD----------
VLSLGKRLIEDE  139

Query  288  RY  289
            Y
Sbjct  140  EY  141
```

>ref|NP_417296.1| exonuclease V (RecBCD complex), alpha chain
[Escherichia coli
str. K-12 substr. MG1655]
 ref|AP_003382.1| exonuclease V (RecBCD complex), alpha chain
[Escherichia coli
str. K-12 substr. W3110]
 ref|YP_001731707.1| exonuclease V (RecBCD complex), alpha chain
[Escherichia coli
str. K-12 substr. DH10B]
 ref|YP_002927750.1| exonuclease V (RecBCD complex), alpha chain
[Escherichia coli
BW2952]
 sp|P04993.2|EX5A_ECOLI RecName: Full=Exodeoxyribonuclease V alpha
chain; AltName: Full=Exodeoxyribonuclease
V 67 kDa polypeptide
 pdb|3K70|D Chain D, Crystal Structure Of The Complete Initiation
Complex
Of Recbcd
 pdb|3K70|G Chain G, Crystal Structure Of The Complete Initiation
Complex
Of Recbcd
 gb|AAB40466.1| exonuclease V alpha-subunit [Escherichia coli str. K-
12 substr.
MG1655]
 gb|AAC75858.1| exonuclease V (RecBCD complex), alpha chain
[Escherichia coli
str. K-12 substr. MG1655]

```
 dbj|BAE76888.1| exonuclease V (RecBCD complex), alpha chain
[Escherichia coli
str. K12 substr. W3110]
 gb|ACB03929.1| exonuclease V (RecBCD complex), alpha chain
[Escherichia coli
str. K-12 substr. DH10B]
 gb|ACR63050.1| exonuclease V (RecBCD complex), alpha chain
[Escherichia coli
BW2952]
Length=608

 Score = 32.3 bits (72),  Expect = 0.062, Method: Compositional matrix
adjust.
 Identities = 18/59 (31%), Positives = 29/59 (49%), Gaps = 0/59 (0%)

Query  146
RYYYSRKVILAYLECALHTDMADIEQYYMKPPGSCFWVAVLDGNVVGIVAARAHEEDNT  204
            R+Y  R V++A  + AL     DI     +  G+  W A+ DGN+  +  +R  E +
T
Sbjct  475
RWYEGRPVMIARNDSALGLFNGDIGIALDRGQGTRVWFAMPDGNIKSVQPSRLPEHETT  533


>gb|AAA97269.1| CG Site No. 277 [Escherichia coli str. K-12 substr.
MG1655]
Length=161

 Score = 30.4 bits (67),  Expect = 0.22, Method: Compositional matrix
adjust.
 Identities = 18/64 (28%), Positives = 31/64 (48%), Gaps = 0/64 (0%)

Query  207
LLRMSVDSRFRGKGIAKALGRRVLEFAMLHNYSAVVLGTTAVKVAAHKLYESLGFRHMGA  266
            L  ++VD  ++ +G+ +AL   +++       + + L   A   AA  LYESLGF
Sbjct  66
LFNIAVDPDYQRQGLGRALLEHLIDELEKRGVATLWLEVRASNAAAIALYESLGFNEATI  125

Query  267  SDHY  270
             +Y
Sbjct  126  RRNY  129


>ref|NP_418790.1| ribosomal-protein-S18-alanine N-acetyltransferase
[Escherichia
coli str. K-12 substr. MG1655]
 ref|AP_004860.1| acetylase for 30S ribosomal subunit protein S18
[Escherichia
coli str. K-12 substr. W3110]
 ref|YP_001733078.1| acetylase for 30S ribosomal subunit protein S18
[Escherichia
coli str. K-12 substr. DH10B]
```

```
 ref|YP_002929255.1| acetylase for 30S ribosomal subunit protein S18
[Escherichia
coli BW2952]
 sp|P0A944.1|RIMI_ECOLI RecName: Full=Ribosomal-protein-alanine
acetyltransferase; AltName:
Full=Acetylating enzyme for N-terminal of ribosomal protein
S18
 gb|AAC77326.1| ribosomal-protein-S18-alanine N-acetyltransferase
[Escherichia
coli str. K-12 substr. MG1655]
 dbj|BAE78361.1| acetylase for 30S ribosomal subunit protein S18
[Escherichia
coli str. K12 substr. W3110]
 gb|ACB05300.1| acetylase for 30S ribosomal subunit protein S18
[Escherichia
coli str. K-12 substr. DH10B]
 gb|ACR61906.1| acetylase for 30S ribosomal subunit protein S18
[Escherichia
coli BW2952]
Length=148

 Score = 30.0 bits (66),  Expect = 0.25, Method: Compositional matrix
adjust.
 Identities = 18/64 (28%), Positives = 31/64 (48%), Gaps = 0/64 (0%)

Query  207
LLRMSVDSRFRGKGIAKALGRRVLEFAMLHNYSAVVLGTTAVKVAAHKLYESLGFRHMGA  266
            L  ++VD  ++ +G+ +AL   +++       + + L   A   AA  LYESLGF
Sbjct  66
LFNIAVDPDYQRQGLGRALLEHLIDELEKRGVATLWLEVRASNAAAIALYESLGFNEATI  125

Query  267  SDHY  270
              +Y
Sbjct  126  RRNY  129


>ref|NP_418210.1| predicted multidrug or homocysteine efflux system
[Escherichia
coli str. K-12 substr. MG1655]
 ref|AP_004033.1| predicted multidrug or homocysteine efflux system
[Escherichia
coli str. K-12 substr. W3110]
 ref|YP_001732573.1| multidrug or homocysteine efflux system
[Escherichia coli str.
K-12 substr. DH10B]
 ref|YP_002928642.1| putative multidrug or homocysteine efflux system
[Escherichia
coli BW2952]
 sp|P31474.1|HSRA_ECOLI RecName: Full=Probable transport protein hsrA;
AltName: Full=High-copy
suppressor of rspA
```

```
 gb|AAC76777.1| predicted multidrug or homocysteine efflux system
[Escherichia
coli str. K-12 substr. MG1655]
 dbj|BAE77534.1| predicted multidrug or homocysteine efflux system
[Escherichia
coli str. K12 substr. W3110]
 gb|ACB04795.1| predicted multidrug or homocysteine efflux system
[Escherichia
coli str. K-12 substr. DH10B]
 gb|ACR64683.1| predicted multidrug or homocysteine efflux system
[Escherichia
coli BW2952]
Length=475

 Score = 29.3 bits (64),  Expect = 0.43, Method: Compositional matrix
adjust.
 Identities = 23/111 (21%), Positives = 49/111 (44%), Gaps = 10/111
(9%)

Query  169
IEQYYMKPPGSCFWVAVLDGNVVGIVAARAHEEDNTVELLRMSVDSRFRGKGIAKALGRR  228
            I Q+ ++ P    W+ +L   ++G+  +      NT+ L  ++ D+   G  +
+
Sbjct  342  IAQFSLQSPAMAIWMLILPLFILGMAMSTQFTAMNTITLADLTDDNASSGNSVLAV--
TQ  399

Query  229  VLEFAMLHNYSAVVL-------GTTAVKVAAHKLYESLGFRHMGASDHYVL  272
             L ++     SA VL       GTT V+   H  + ++G   + ++  ++L
Sbjct  400  QLSISLGVAVSAAVLRVYEGMEGTTTVE-QFHYTFITMGIITVASAAMFML  449


>ref|NP_417625.1| predicted acyltransferase with acyl-CoA N-
acyltransferase domain
[Escherichia coli str. K-12 substr. MG1655]
 ref|AP_003701.1| predicted acyltransferase with acyl-CoA N-
acyltransferase domain
[Escherichia coli str. K-12 substr. W3110]
 ref|YP_001732013.1| acyltransferase [Escherichia coli str. K-12
substr. DH10B]
 ref|YP_002928056.1| putative acyltransferase with acyl-CoA N-
acyltransferase domain
[Escherichia coli BW2952]
 sp|P63417.1|YHBS_ECOLI RecName: Full=Uncharacterized N-
acetyltransferase YhbS
 gb|AAA57959.1| ORF_f167; end overlaps end of o100 by 14 bases; start
overlaps
f174, other starts possible [Escherichia coli str. K-12 substr.
MG1655]
 gb|AAC76190.1| predicted acyltransferase with acyl-CoA N-
acyltransferase domain
[Escherichia coli str. K-12 substr. MG1655]
```

dbj|BAE77202.1| predicted acyltransferase with acyl-CoA N-acyltransferase domain
[Escherichia coli str. K12 substr. W3110]
gb|ACB04235.1| predicted acyltransferase with acyl-CoA N-acyltransferase domain
[Escherichia coli str. K-12 substr. DH10B]
gb|ACR64126.1| predicted acyltransferase with acyl-CoA N-acyltransferase domain
[Escherichia coli BW2952]
Length=167

Score = 28.9 bits (63), Expect = 0.58, Method: Compositional matrix adjust.
Identities = 27/81 (33%), Positives = 39/81 (48%), Gaps = 12/81 (15%)

```
Query  187  DGNVVGIVA---ARAHEED-
NTVELLRMSVDSRFRGKGIAKALGRRVLEFAMLHNYSAVV  242
            +G V+G VA        ED   V +  ++VD ++RG+G+A+ L    L+
Y+AVV
Sbjct  50
EGQVIGYVAFSPVDVQGEDLQWVGMAPLAVDEKYRGQGLARQLVYEGLDSLNEFGYAAVV  109

Query  243  -LGTTAVKVAAHKLYESLGFR  262
             LG  A       LY   GF
Sbjct  110  TLGDPA-------LYSRFGFE  123
```

>ref|NP_417454.1| DNA-binding transcriptional dual regulator, glycolate-binding
[Escherichia coli str. K-12 substr. MG1655]
ref|AP_003535.1| DNA-binding transcriptional dual regulator, glycolate-binding
[Escherichia coli str. K-12 substr. W3110]
ref|YP_001731848.1| DNA-binding transcriptional dual regulator, glycolate-binding
[Escherichia coli str. K-12 substr. DH10B]
ref|YP_002927895.1| DNA-binding transcriptional dual regulator, glycolate-binding
[Escherichia coli BW2952]
sp|P0ACL5.1|GLCC_ECOLI RecName: Full=Glc operon transcriptional activator
gb|AAB02529.1| regulatory protein [Escherichia coli str. K-12 substr. W3110]
gb|AAC76016.1| DNA-binding transcriptional dual regulator, glycolate-binding
[Escherichia coli str. K-12 substr. MG1655]
dbj|BAE77041.1| DNA-binding transcriptional dual regulator, glycolate-binding
[Escherichia coli str. K12 substr. W3110]
gb|ACB04070.1| DNA-binding transcriptional dual regulator, glycolate-binding
[Escherichia coli str. K-12 substr. DH10B]

gb|ACR62941.1| DNA-binding transcriptional dual regulator, glycolate-binding
[Escherichia coli BW2952]
Length=254

Score = 27.3 bits (59), Expect = 1.8, Method: Compositional matrix adjust.
Identities = 10/23 (43%), Positives = 15/23 (65%), Gaps = 0/23 (0%)

```
Query  92   FYDGILERIPNTAFRGLRQHPRT  114
             Y+ +L+R+P+ A R  R H RT
Sbjct  205  IYNAVLQRLPHVAQRAARDHVRT  227
```

>gb|AAA69147.1| ORF_o274 [Escherichia coli str. K-12 substr. MG1655]
Length=274

Score = 27.3 bits (59), Expect = 2.0, Method: Compositional matrix adjust.
Identities = 10/23 (43%), Positives = 15/23 (65%), Gaps = 0/23 (0%)

```
Query  92   FYDGILERIPNTAFRGLRQHPRT  114
             Y+ +L+R+P+ A R  R H RT
Sbjct  225  IYNAVLQRLPHVAQRAARDHVRT  247
```

>ref|YP_001730194.1| ATP-binding subunit of oligopeptide ABC transporter [Escherichia
coli str. K-12 substr. DH10B]
ref|YP_002926275.1| oligopeptide transporter subunit [Escherichia coli BW2952]
gb|ACB02416.1| ATP-binding subunit of oligopeptide ABC transporter [Escherichia
coli str. K-12 substr. DH10B]
gb|ACR63934.1| oligopeptide transporter subunit [Escherichia coli BW2952]
Length=334

Score = 26.6 bits (57), Expect = 2.8, Method: Compositional matrix adjust.
Identities = 13/50 (26%), Positives = 27/50 (54%), Gaps = 5/50 (10%)

```
Query  151  RKVILAYLECALHTDMADIEQYYMKPPGSCFWVA-----VLDGNVVGIVA  195
            RKV+L   +  +H ++ D +Q++ +PP +   V      + +G  +G+V
Sbjct  8    RKVLLEIADLKVHFEIKDGKQWFWQPPKTLKAVDGVTLRLYEGETLGVVG  57
```

>ref|NP_415763.1| oligopeptide transporter subunit [Escherichia coli str. K-12
substr. MG1655]
ref|AP_001873.1| oligopeptide transporter subunit [Escherichia coli str. K-12

substr. W3110]
sp|P77737.1|OPPF_ECOLI RecName: Full=Oligopeptide transport ATP-binding protein OppF
dbj|BAA14779.1| oligopeptide transporter subunit [Escherichia coli str. K12 substr.
W3110]
gb|AAC74329.1| oligopeptide transporter subunit [Escherichia coli str. K-12
substr. MG1655]
Length=334

Score = 26.6 bits (57), Expect = 2.8, Method: Compositional matrix adjust.
Identities = 13/50 (26%), Positives = 27/50 (54%), Gaps = 5/50 (10%)

```
Query  151  RKVILAYLECALHTDMADIEQYYMKPPGSCFWVA-----VLDGNVVGIVA  195
            RKV+L   +  +H ++ D +Q++ +PP +   V      + +G  +G+V
Sbjct  8    RKVLLEIADLKVHFEIKDGKQWFWQPPKTLKAVDGVTLRLYEGETLGVVG  57
```

>ref|NP_416506.1| CP4-44 prophage; predicted DNA repair protein [Escherichia coli
str. K-12 substr. MG1655]
ref|AP_002601.1| predicted DNA repair protein [Escherichia coli str. K-12 substr.
W3110]
ref|YP_001730951.1| CP4-44 prophage; DNA repair protein [Escherichia coli str. K-12
substr. DH10B]
ref|YP_002926989.1| CP4-44 prophage; putative DNA repair protein [Escherichia coli
BW2952]
sp|P76362.1|YEES_ECOLI RecName: Full=UPF0758 protein yeeS
dbj|BAA15826.1| predicted DNA repair protein [Escherichia coli str. K12 substr.
W3110]
gb|AAC75063.1| CP4-44 prophage; predicted DNA repair protein [Escherichia coli
str. K-12 substr. MG1655]
gb|ACB03173.1| CP4-44 prophage; predicted DNA repair protein [Escherichia coli
str. K-12 substr. DH10B]
gb|ACR62614.1| CP4-44 prophage; predicted DNA repair protein [Escherichia coli
BW2952]
Length=148

Score = 26.6 bits (57), Expect = 2.9, Method: Compositional matrix adjust.
Identities = 19/66 (29%), Positives = 32/66 (48%), Gaps = 11/66 (17%)

```
Query  227  RRVLEFAMLHNYSAVVLG----------
TTAVKVAAHKLYESLGFRHMGASDHYVLPG-M  275
            R V++ A+ HN +AVVL           + A ++    +L ++LG   +    DH ++ G
Sbjct  79
REVIKRALYHNAAAVVLAHNHPSGEVTPSKADRLITERLVQALGLVDIRVPDHLIVGGNQ  138

Query  276  TLSLAE  281
              S AE
Sbjct  139  VFSFAE  144
```

>ref|NP_418517.1| predicted acyltransferase with acyl-CoA N-acyltransferase domain
[Escherichia coli str. K-12 substr. MG1655]
 ref|AP_004595.1| predicted acyltransferase with acyl-CoA N-acyltransferase domain
[Escherichia coli str. K-12 substr. W3110]
 ref|YP_001732867.1| acyltransferase [Escherichia coli str. K-12 substr. DH10B]
 ref|YP_002928999.1| putative acyltransferase with acyl-CoA N-acyltransferase domain
[Escherichia coli BW2952]
 sp|P16691.1|PHNO_ECOLI RecName: Full=Protein PhnO
 dbj|BAA14275.1| phnO [Escherichia coli W3110]
 gb|AAA96992.1| phnO [Escherichia coli str. K-12 substr. MG1655]
 gb|AAC77054.1| predicted acyltransferase with acyl-CoA N-acyltransferase domain
[Escherichia coli str. K-12 substr. MG1655]
 dbj|BAE78096.1| predicted acyltransferase with acyl-CoA N-acyltransferase domain
[Escherichia coli str. K12 substr. W3110]
 gb|ACB05089.1| predicted acyltransferase with acyl-CoA N-acyltransferase domain
[Escherichia coli str. K-12 substr. DH10B]
 gb|ACR64127.1| predicted acyltransferase with acyl-CoA N-acyltransferase domain
[Escherichia coli BW2952]
Length=144

 Score = 25.8 bits (55), Expect = 5.4, Method: Compositional matrix adjust.
 Identities = 22/92 (24%), Positives = 37/92 (40%), Gaps = 4/92 (4%)

```
Query  174  MKPPGSCFWVAVLDGNVVGIVAA----
RAHEEDNTVELLRMSVDSRFRGKGIAKALGRRV  229
            ++ P   + +A+LDG VVG++        H  +   E+  + V  + RG  +   L
Sbjct  42
LRDPNMRYHLALLDGEVVGMIGLHLQFHLHHVNWIGEIQELVVMPQARGLNVGSKLLAWA  101

Query  230  LEFAMLHNYSAVVLGTTAVKVAAHKLYESLGF  261
             E A         L T   +  AH+ Y   G+
Sbjct  102  EEEARQAGAEMTELSTNVKRHDAHRFYLREGY  133
```

```
>ref|AP_004342.1| glycerol-3-phosphate transporter subunit
[Escherichia coli str.
K-12 substr. W3110]
 ref|NP_417907.2| glycerol-3-phosphate transporter subunit
[Escherichia coli str.
K-12 substr. MG1655]
 ref|YP_001732284.1| glycerol-3-phosphate ABC transporter ATP-binding
protein [Escherichia
coli str. K-12 substr. DH10B]
 ref|YP_002928337.1| glycerol-3-phosphate transporter subunit
[Escherichia coli BW2952]
 sp|P10907.3|UGPC_ECOLI RecName: Full=sn-glycerol-3-phosphate import
ATP-binding protein
UgpC
 dbj|BAE77843.1| glycerol-3-phosphate transporter subunit [Escherichia
coli str.
K12 substr. W3110]
 gb|AAC76475.2| glycerol-3-phosphate transporter subunit [Escherichia
coli str.
K-12 substr. MG1655]
 gb|ACB04506.1| glycerol-3-phosphate transporter subunit; ATP-binding
component
of ABC superfamily [Escherichia coli str. K-12 substr. DH10B]
 gb|ACR63359.1| glycerol-3-phosphate transporter subunit [Escherichia
coli BW2952]
Length=356

 Score = 25.0 bits (53),  Expect = 7.7, Method: Compositional matrix
adjust.
 Identities = 14/38 (37%), Positives = 21/38 (55%), Gaps = 0/38 (0%)

Query  73   RGVCIREFRAAEQEAARRIFYDGILERIPNTAFRGLRQ  110
            RG+  ++     +EAAR +  DG+L+R P     G RQ
Sbjct  104  RGMGKQQIAERVKEAARILELDGLLKRRPRELSGGQRQ  141


>ref|AP_002454.1| hypothetical protein [Escherichia coli str. K-12
substr. W3110]
 ref|NP_416348.2| conserved protein [Escherichia coli str. K-12
substr. MG1655]
 ref|YP_001730810.1| hypothetical protein ECDH10B_1973 [Escherichia
coli str. K-12
substr. DH10B]
 ref|YP_002926845.1| hypothetical protein BWG_1648 [Escherichia coli
BW2952]
 sp|P76272.2|YEBT_ECOLI RecName: Full=Uncharacterized protein yebT
 dbj|BAA15647.1| conserved hypothetical protein [Escherichia coli str.
K12 substr.
W3110]
```

gb|AAC74904.2| conserved protein [Escherichia coli str. K-12 substr. MG1655]
gb|ACB03032.1| conserved protein [Escherichia coli str. K-12 substr. DH10B]
gb|ACR62492.1| conserved protein [Escherichia coli BW2952]
Length=877

Score = 25.0 bits (53), Expect = 7.8, Method: Compositional matrix adjust.
Identities = 15/59 (25%), Positives = 30/59 (51%), Gaps = 9/59 (15%)

```
Query  158  LECALHTDMAD-----IEQYYMKPPGSCFWVAVLD----
GNVVGIVAARAHEEDNTVEL  207
            ++ ++ +DM D      + + + P  S   V+ LD    GN +G++  +  E+D+ V
L
Sbjct  90
VKVSIKSDMKDALREETQFWLVTPKASLAGVSGLDALVGGNYIGMMPGKGKEQDHFVAL  148
```

>gb|AAB18425.1| CG Site No. 17794 [Escherichia coli str. K-12 substr. MG1655]
Length=369

Score = 25.0 bits (53), Expect = 7.9, Method: Compositional matrix adjust.
Identities = 14/38 (37%), Positives = 21/38 (55%), Gaps = 0/38 (0%)

```
Query  73   RGVCIREFRAAEQEAARRIFYDGILERIPNTAFRGLRQ  110
            RG+  ++     +EAAR +  DG+L+R P     G RQ
Sbjct  117  RGMGKQQIAERVKEAARILELDGLLKRRPRELSGGQRQ  154
```

>ref|NP_417259.1| enolase [Escherichia coli str. K-12 substr. MG1655]
ref|AP_003345.1| enolase [Escherichia coli str. K-12 substr. W3110]
ref|YP_001731671.1| enolase [Escherichia coli str. K-12 substr. DH10B]
ref|YP_002927711.1| enolase [Escherichia coli BW2952]
sp|P0A6P9.2|ENO_ECOLI RecName: Full=Enolase; AltName: Full=2-phospho-D-glycerate hydro-lyase;
AltName: Full=2-phosphoglycerate dehydratase
sp|B1XDI9.1|ENO_ECODH RecName: Full=Enolase; AltName: Full=2-phospho-D-glycerate hydro-lyase;
AltName: Full=2-phosphoglycerate dehydratase
sp|C4ZZT2.1|ENO_ECOBW RecName: Full=Enolase; AltName: Full=2-phospho-D-glycerate hydro-lyase;
AltName: Full=2-phosphoglycerate dehydratase
gb|AAA69289.1| enolase [Escherichia coli str. K-12 substr. MG1655]
gb|AAC75821.1| enolase [Escherichia coli str. K-12 substr. MG1655]
dbj|BAE76853.1| enolase [Escherichia coli str. K12 substr. W3110]
gb|ACB03893.1| enolase [Escherichia coli str. K-12 substr. DH10B]
gb|ACR63031.1| enolase [Escherichia coli BW2952]
Length=432

Score = 25.0 bits (53), Expect = 9.2, Method: Compositional matrix adjust.
Identities = 17/42 (40%), Positives = 23/42 (55%), Gaps = 2/42 (5%)

```
Query  186  LDGNVVGIVAARAHEEDNTVELL--RMSVDSRFRGKGIAKAL  225
            L+G  VG+ AA +     + E L  R    SRF GKG+ KA+
Sbjct  27   LEGGFVGMAAAPSGASTGSREALELRDGDKSRFLGKGVTKAV  68
```

>ref|NP_418334.1| predicted peptidase [Escherichia coli str. K-12 substr. MG1655]
ref|AP_003910.1| predicted endo-1,4-beta-glucanase [Escherichia coli str. K-12
substr. W3110]
ref|YP_001732690.1| endo-1,4-beta-glucanase [Escherichia coli str. K-12 substr. DH10B]
ref|YP_002928761.1| putative endo-1,4-beta-glucanase [Escherichia coli BW2952]
sp|P32153.1|FRVX_ECOLI RecName: Full=Putative aminopeptidase frvX
gb|AAB03031.1| ORF_f356 [Escherichia coli str. K-12 substr. MG1655]
gb|AAC76880.1| predicted peptidase [Escherichia coli str. K-12 substr. MG1655]
dbj|BAE77411.1| predicted endo-1,4-beta-glucanase [Escherichia coli str. K12
substr. W3110]
gb|ACB04912.1| predicted endo-1,4-beta-glucanase [Escherichia coli str. K-12
substr. DH10B]
gb|ACR64341.1| predicted endo-1,4-beta-glucanase [Escherichia coli BW2952]
Length=356

Score = 25.0 bits (53), Expect = 9.4, Method: Compositional matrix adjust.
Identities = 9/25 (36%), Positives = 15/25 (60%), Gaps = 0/25 (0%)

```
Query  142  LLALRYYYSRKVILAYLECALHTDM  166
            L   RY+ ++K++ A   CA H D+
Sbjct  255  LFDKRYFPNQKLVAALKSCAAHNDL  279
```

>ref|NP_416307.1| conserved outer membrane lipoprotein [Escherichia coli str. K-12
substr. MG1655]
ref|AP_002412.1| conserved outer membrane protein [Escherichia coli str. K-12
substr. W3110]
ref|YP_001730769.1| outer membrane protein [Escherichia coli str. K-12 substr. DH10B]
ref|YP_002926803.1| conserved outer membrane protein [Escherichia coli BW2952]

```
 sp|P64493.1|YOAF_ECOLI RecName: Full=Uncharacterized protein yoaF
 gb|AAC74863.1| conserved outer membrane lipoprotein [Escherichia coli
str. K-12
substr. MG1655]
 dbj|BAE76530.1| conserved outer membrane protein [Escherichia coli
str. K12 substr.
W3110]
 gb|ACB02991.1| conserved outer membrane protein [Escherichia coli
str. K-12
substr. DH10B]
 gb|ACR62258.1| conserved outer membrane protein [Escherichia coli
BW2952]
Length=84

 Score = 25.0 bits (53),  Expect = 9.7, Method: Composition-based
stats.
 Identities = 16/37 (43%), Positives = 17/37 (46%), Gaps = 3/37 (8%)

Query  31  LLVAAGAMWPPLPAAPGP---AAAPPPAAGPQPHGGT  64
           LLV AG   P  P AP P     A P +   Q  GGT
Sbjct  11  LLVLAGCSTPSQPEAPKPPQIGMANPASVYCQQKGGT  47


  Database: All non-redundant GenBank CDS
translations+PDB+SwissProt+PIR+PRF
excluding environmental samples from WGS projects
    Posted date:  Mar 22, 2011  4:36 PM
  Number of letters in database: 1,896,392
  Number of sequences in database:  6,139

Lambda     K      H
   0.324    0.139    0.429
Gapped
Lambda     K      H
   0.267   0.0410    0.140
Matrix: BLOSUM62
Gap Penalties: Existence: 11, Extension: 1
Number of Sequences: 6139
Number of Hits to DB: 14580
Number of extensions: 649
Number of successful extensions: 0
Number of sequences better than 100: 0
Number of HSP's better than 100 without gapping: 0
Number of HSP's gapped: 0
Number of HSP's successfully gapped: 0
Length of query: 299
Length of database: 1896392
Length adjustment: 85
Effective length of query: 214
Effective length of database: 1374577
Effective search space: 294159478
Effective search space used: 294159478
```

T: 11
A: 40
X1: 15 (7.0 bits)
X2: 38 (14.6 bits)
X3: 64 (24.7 bits)
S1: 40 (20.0 bits)
S2: 44 (21.6 bits)
